# The 3xTg‐AD Mouse Model of Alzheimer's Disease Exhibits Lifelong Reductions in Circulating Choline Despite Adequate Dietary Intake, With Sex‐Specific Neuropathological and Behavioral Phenotypes

**DOI:** 10.1111/acel.70330

**Published:** 2025-12-29

**Authors:** Jessica M. Judd, Faizan Mistry, Wendy Winslow, Savannah Tallino, Julie Turk, Ramon Velazquez

**Affiliations:** ^1^ Arizona State University‐Banner Neurodegenerative Disease Research Center at the Biodesign Institute Arizona State University Tempe Arizona USA; ^2^ School of Life Sciences Arizona State University Tempe Arizona USA

**Keywords:** 3xTg‐AD mice, aging, behavior, choline, neuropathology, sex differences

## Abstract

Alzheimer's disease (AD) is a neurodegenerative disease characterized by amyloid‐beta plaques and neurofibrillary tau tangles in the brain, neuroinflammation, and cognitive impairment. The 3xTg‐AD mouse is a commonly used model in AD studies. 3xTg‐AD males display inconsistent pathology; therefore, most studies utilize females. An understanding of why sexual dimorphism exists in this model is lacking. In humans, low circulating choline levels are associated with elevated AD pathology, while higher choline intake reduces pathology in AD mouse models. Here, we sought to understand if blood choline levels are associated with the sex discrepancies observed in 3xTg‐AD mice. Body weight and chow consumption were measured, and blood plasma samples were collected at 3, 6, 9, 12 months of age and at end‐point in 3xTg‐AD and NonTg mice. 3xTg‐AD females and NonTg males consumed more chow and gained more body weight than other groups. Longitudinally, 3xTg‐AD mice had lower plasma choline levels than NonTg mice, while levels declined with age in NonTg mice. Female 3xTg‐AD mice had higher AD‐like pathological burden than males, but males had higher mortality rates across the study. IntelliCage automated phenotyping revealed high water‐seeking behavior in males. 3xTg‐AD mice displayed higher impulsivity compared to NonTg mice. Males were better at spatial and attention tasks but perseverated during avoidance testing compared with females. These findings demonstrate a persistent reduction in circulating choline levels across the lifespan of 3xTg‐AD mice despite adequate dietary intake. Given choline's roles in metabolism, inflammatory regulation, and neuronal function, chronically low circulating choline may contribute to the various dysfunctions observed in this model.

## Graphical abstract

This study found that 3xTg‐AD mice exhibited lifelong reductions in circulating choline. Female 3xTg‐AD mice showed greater AD‐like pathology, whereas males displayed higher mortality, impulsivity, and water‐seeking behavior. These sex‐specific differences suggest distinct metabolic and behavioral vulnerabilities contributing to AD progression in this model.

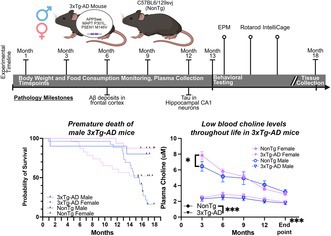

## 1 Introduction

Alzheimer's disease (AD) is an age‐related neurodegenerative disease and the most common form of dementia; AD currently afflicts 6.7 million people aged 65 and over in the United States (Alzheimer's Association 2025). Women are more frequently diagnosed with AD than men and account for two‐thirds of cases (Alzheimer's Association 2025). The hallmark clinical symptoms of AD include progressive memory loss and executive dysfunction (Scheltens et al. 2021; Alzheimer's Association 2025). As AD progresses, impairments in basic functions—such as swallowing—develop, ultimately resulting in death (Scheltens et al. 2021; Alzheimer's Association 2025). The clinical symptoms of AD are driven by the accumulation of amyloid beta (Aβ) plaques and neurofibrillary tau tangles (NFTs), neuroinflammation, and neuronal loss (Scheltens et al. 2021; Alzheimer's Association 2025). While therapies have been developed, including an FDA‐approved drug that targets Aβ plaques (Cummings et al. 2023), there is an urgent need to understand mechanisms contributing to AD pathogenesis to enable the development of both preventive and therapeutic strategies to halt other dominant pathologies.

The triple transgenic mouse (3xTg‐AD) is a commonly used rodent model of AD that harbors familial human mutations amyloid precursor protein (APP Swedish) and microtubule associated protein tau (MAPT P301L), and a presenilin 1 knock‐in mutation (PSEN1 M146V) (Oddo et al. 2003; Winslow et al. 2021). These mutations result in AD‐like pathology: by 6 months of age, Aβ pathology develops in the frontal cortex and there are elevations in pathological tau hyperphosphorylation in the hippocampus, and by 12 months of age pathology is widespread (Oddo et al. 2003; Winslow et al. 2021). Additionally, these mice display behavioral deficits that parallel AD's clinical presentation: spatial learning and memory deficits can be detected as early as 6 months and become progressively worse by 12 months (Winslow et al. 2021; Roda et al. 2020; Parachikova et al. 2010). However, 3xTg‐AD mice show notable sex differences; males inconsistently develop pathology, but they are more prone to frailty and earlier mortality (Dennison et al. 2021; Barber et al. 2024), which is similar to mortality sex differences observed in humans (reviewed in Mielke et al. (2014)). This recapitulation of human disease manifestation has made the 3xTg‐AD mouse an invaluable tool for studying the mechanisms underlying AD and for preclinical assessment of potential AD interventions and therapeutics.

An understudied factor that has recently gained attention due to its association with AD risk and pathological progression is choline, an essential nutrient that plays important roles in maintaining health across multiple organ systems, including the brain (Dave et al. 2023; Institute of Medicine 1998). Choline demand is met by both endogenous production and dietary intake. Endogenous choline production by the phosphatidylethanolamine N‐methyltransferase (PEMT) enzyme in the liver satisfies about 30% of the required choline (Institute of Medicine 1998; Fischer et al. 2007), while the remaining 70% must be acquired through diet. The Institute of Medicine set guidelines for dietary intake in 1998 of 550 and 425 mg/day for adult men and women, respectively (Institute of Medicine 1998). These guidelines were set to prevent nonalcoholic fatty liver disease (Institute of Medicine 1998). Alarmingly, it is estimated that only 11% of Americans achieve an adequate daily intake of choline (Wallace and Fulgoni 2016) and similar deficiencies are observed worldwide (Vennemann et al. 2015). Further, genetic differences can alter the function of the PEMT enzyme and endogenous choline production (Korbecki et al. 2024). Polymorphisms of PEMT have been associated with obesity, metabolic disturbances, cardiovascular disease, liver disease, and AD (Bi et al. 2012; Institute of Medicine 1998; Korbecki et al. 2024). Choline is a precursor for several molecules throughout the brain and body, including for phospholipids that compose cell membranes and for the neurotransmitter acetylcholine (ACh) (Blusztajn 1998). As a precursor to the methyl donor betaine, choline is important in epigenetic regulation and in the conversion of homocysteine to methionine; homocysteine is toxic when levels become elevated (Dymek et al. 2024; Blusztajn 1998). Low choline levels can result in liver pathology, cardiovascular disease, and metabolism dysfunction (Dave et al. 2023; Institute of Medicine 1998). Choline is also vital for brain health across the lifespan. Maternal choline supplementation during gestation promotes nervous system development and improves both cognitive function throughout life and metabolic processes (Dymek et al. 2024). Choline is also protective against cognitive decline and neurodegeneration (Nurk et al. 2013; Yuan et al. 2022; Liu et al. 2014), and low circulating choline levels correlate with AD pathological severity and neuroinflammation; the lowest levels corresponded with the highest AD pathological burden (Judd et al. 2023). Further, reduced ACh resulting from loss of cholinergic neurons is an established feature of AD (Schliebs and Arendt 2011). Rodent studies are consistent with these findings in humans and show that a diet deficient in choline throughout the lifespan results in reduced circulating choline and exacerbated AD pathology (Judd et al. 2023; Dave et al. 2023). Supplementation of choline above the recommended daily value through adulthood is protective, reducing AD‐like pathology and neuroinflammation (Judd et al. 2023; Velazquez et al. 2019; Wang et al. 2019). Taken together, adequate choline levels are essential for health across multiple organ systems and are particularly important for brain health.

Interestingly, we recently found that, at 7 months of age—prior to extensive pathology—plasma choline levels are lower in 3xTg‐AD female mice than in their non‐transgenic (NonTg) counterparts (Judd et al. 2023). Further, 3xTg‐AD mice, but not NonTg mice, showed signs of organ pathologies associated with low choline, even when maintained on an adequate choline diet, suggesting potential impairments with endogenous choline production. Notably, 3xTg‐AD mice consume more chow than NonTg and, therefore, should ingest more total choline than other groups; however, this is not reflected in circulating plasma levels (Dave et al. 2023). Together, these findings suggest that 3xTg‐AD mice may require substantially more choline intake than NonTg to compensate for metabolic dysfunction inherent in this model. Thus, the goal of this study was to determine whether sex discrepancies in pathological trajectory in 3xTg‐AD mice are associated with varying circulating choline levels. Unlike 3xTg‐AD females, 3xTg‐AD males do not show consistent pathological development (Dave et al. 2023; Winslow et al. 2021; Kapadia et al. 2018; Dennison et al. 2021). Consequently, assessing choline levels through the lifespan of male and female 3xTg‐AD mice and measuring the subsequent development of AD pathology may give further insight into the 3xTg‐AD mouse model and the role endogenous choline plays in AD pathological development. We measured body weight and chow consumption throughout the lifespan and collected blood plasma at multiple time points. We then conducted a behavior battery to assess a variety of cognitive domains. Hippocampal and cortical tissue were analyzed for markers of Aβ and tau pathology. We hypothesize that males and females would show discrepancies in choline levels that correspond to divergent pathological outcomes.

## 2 Materials and Methods

### 2.1 Animals

3xTg‐AD homozygous mice were generated on a C57BL6/129Svj hybrid background, as previously described (Winslow et al. 2021; Judd et al. 2023; Dave et al. 2023; Velazquez et al. 2017). For NonTg controls, C57BL6/129Svj mice were used. All procedures were approved in advance by the Institutional Animal Care and Use Committee of Arizona State University. Both male and female mice were used in this study. Mice were kept on a 12‐h light/dark cycle at 23°C with *ad libitum* access to chow and water, except where noted. All mice were provided a standard laboratory diet (Teklad 2018, Inotiv). Mice were group housed with up to five mice per cage.

### 2.2 Study Timeline

**FIGURE 1:**
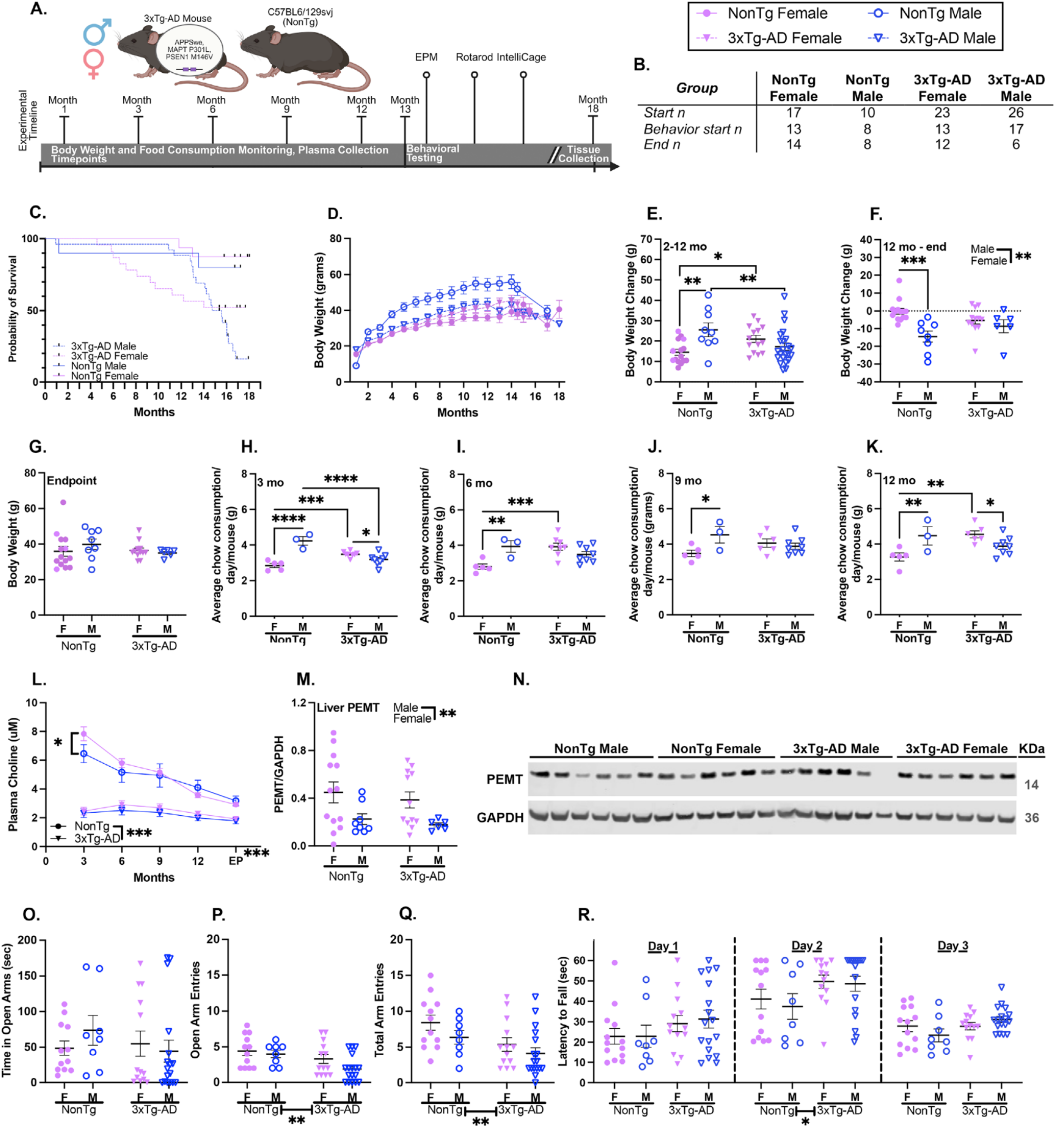
Body weight, chow consumption, and plasma choline levels were measured across the lifespan. (A) Timeline showing when measurements were taken. (B) Table of the number of mice in each group at various stages of the experiment. (C) Survival curve for all groups showing high attrition by 3xTg‐AD mice. (D) Mice gained body weight until 12 months of age but dropped in body weight afterwards. (E) Between 2 and 12 months, 3xTg‐AD females gained more weight than NonTg females, and 3xTg‐AD males gained less weight than NonTg males. (F) Between 12 months and endpoint, males lost more weight than females. (G) At endpoint, there were no differences in body weight between the groups. (H) Each data point is an average of food consumption per cage. At 3 months, 3xTg‐AD females and NonTg males consumed more than 3xTg‐AD males and NonTg females. (I) At 6 months, 3xTg‐AD females and NonTg males consumed more than NonTg females. (J) At 9 months, NonTg males consumed more than NonTg females. (K) At 12 months, 3xTg‐AD females consumed more than 3xTg‐AD males and NonTg females. NonTg males consumed more than NonTg females. (L) NonTg male mice had lower plasma choline levels than NonTg females at 3 months of age. From 3 months of age to the end point (EP) measure, 3xTg‐AD mice had lower plasma choline levels than NonTg mice. Choline levels declined with age. (M) Quantitative analysis of the PEMT protein from liver homogenates shows an increase in female mice regardless of genotype. (N) Representative western blot for PEMT and loading control GAPDH. For Elevated Plus Maze (EPM), (O) there were no group differences for time in open arms. There were more open arm entries (P) and total arm entries (Q) made by NonTg than 3xTg‐AD. (R) On Day 2 of the Rotarod, 3xTg‐AD had a higher latency to fall (more time on the spinning rod) than NonTg, but groups were similar on Days 1 and 3. Data are reported as means ± SEM. *p < 0.05, **p < 0.01, ***p < 0.001, ****p < 0.0001.

Figure 1A illustrates the timeline of the study. At 3, 6, 9, and 12 months of age, body weight, and chow consumption were monitored, and blood plasma was collected. At endpoint, body weight was measured, and blood plasma was collected. The four groups in this study were NonTg female (starting *n* = 17), NonTg male (starting *n* = 10), 3xTg‐AD female (starting *n* = 23), and 3xTg‐AD male (starting *n* = 26). The number of mice utilized for the study was consistent with prior reports using the 3xTg‐AD model (Dave et al. 2023; Judd et al. 2024; Winslow et al. 2021). Mortality reduced the number of mice that underwent behavioral testing and that survived to the end of the study (Figure 1B). Starting at 13.48 ± 0.12 months (range: 12–14 months), mice underwent behavioral testing. All mice alive at this time participated in behavioral testing, except for one NonTg female who had a missing eye at birth but was otherwise healthy. The behavioral battery took place across 2.5 months. Mice underwent Elevated Plus Maze (EPM), Rotarod, and IntelliCage testing, which assess anxiety‐like behavior, motor ability and learning, and spatial learning and cognitive flexibility, respectively. A week after the end of IntelliCage testing, mice were euthanized, and tissue was harvested.

### 2.3 Body Weight Measurement and Chow Consumption

Body weights were measured throughout the study. To assess chow consumption, equal quantities of chow (grams) were added to each cage on the first day of assessment and then weighed every 24 h for the next 6 days to assess chow consumed each day/number of mice in a cage. Mice remained group housed with their established cage mates during this assessment as previously described (Dave et al. 2023).

### 2.4 Behavior

#### 2.4.1 Elevated Plus Maze (EPM)

Mice underwent EPM testing to assess anxiety‐like behavior (Nishimura et al. 2017). The EPM has two open arms and two enclosed arms which are perpendicular to each other. The apparatus (Panlab) is 650 (W) × 650 (D) mm, and each arm is 60 (W) × 295 (D) × 550 (H, closed arm) mm. The maze is elevated 690 mm from the floor. Each mouse underwent a 3‐min trial, where they were able to freely explore the maze. Open‐ and closed‐arm entries and time spent in open arms were recorded via EthoVisionXT (Noldus Information Technology, v14.0).

#### 2.4.2 Rotarod

Three days after EPM, mice underwent 3 days of Rotarod testing to assess motor coordination and endurance (Dave et al. 2023). The Rotarod apparatus (TSE Systems) contains a rotating rod and floor sensors to detect when mice fall off the rod (detecting latency to fall). The system is connected to a computer with software (TSE Rotarod, version 5.1.2, TSE Systems) that reports latency to fall. The first 2 days were training days, and the third day was a probe test. Each day consisted of five 60‐s trials. During the training days, the rod accelerated at 0.75 rotations per minute (rpm) for 20 s until it reached 15 rpm, which was maintained for 40 s. During the probe test day, the rod accelerated at a steady increase of 1 rpm/s for up to 60 s (max 60 rpm).

#### 2.4.3 IntelliCage

Approximately 2.5 weeks after Rotarod, IntelliCage testing began and continued for 46 days to assess a variety of behaviors, including water seeking, exploratory behavior, spatial learning, reference memory, behavioral flexibility, impulsivity and attention (Masuda et al. 2016, 2018; Mifflin et al. 2021; Winslow et al. 2021; Judd et al. 2024; Tallino et al. 2024). The IntelliCage procedure was based on our previously described protocols (Mifflin et al. 2021; Winslow et al. 2021; Judd et al. 2024; Tallino et al. 2024). A radiofrequency identification transponder (RFID; Standard Microchip T‐VA, DataMars, Switzerland, and Troven, USA) chip was subcutaneously implanted into the dorso‐cervical region under isoflurane inhalation anesthesia 16 days before introduction to the IntelliCage (Winslow et al. 2021; Mifflin et al. 2021). Mice began testing at ~14.15 months of age. Our lab can test 64 mice at a time; we have four IntelliCages and each can house up to 16 mice. Cages were closely monitored for aggression because mice that had not been previously housed together were combined in the IntelliCage. Mice were separated by sex, but each cage was balanced for genotype.

The IntelliCage apparatus (39 (D) × 58 (W) × 21 (H) cm) has four operant corners that use water as a reinforcer (Masuda et al. 2016, 2018; Mifflin et al. 2021; Winslow et al. 2021). Corners are accessible through a tunnel with an antenna that scans a mouse's RFID to register its presence. For a visit to be counted, an animal must fully enter a corner. Sensors on the nose port and waterspout detect nose pokes and licks at each corner. A computer management system is used to control the automated doors and access to water, and to record the actions of the mice in the corners (TSE IntelliCagePlus Analyzer software, Version 3.5.0.0, TSE systems). Mice had *ad libitum* access to standard rodent chow throughout IntelliCage testing. The IntelliCage was filled with Diamond Dry bedding. Lights were on in the behavior room from 06:00 to 18:00. The sequence of experimental behavioral tasks in the IntelliCage were as follows: (1) adaptation phases; (2) place preference and reversal; (3) reaction time tasks with varying pre‐cue delays and cue onsets; and (4) place avoidance and extinction. Mice were monitored for engagement and if they did not consume water within a 24‐h period, they were allowed to access water *ad libitum* in a standard mouse cage for 4 h. Any mouse that failed to access water via the IntelliCage for three consecutive days for any reason (lack of motivation, declining health) was excluded from further testing.

Data were extracted using the TSE IntelliCagePlus Analyzer software and exported to multiple tab‐delimited text files. A Python script converted the data into a single SQLite3 database file as previously described (Winslow et al. 2021; Judd et al. 2024). The SQL database was queried using Python scripts to extract relevant dependent variables that were sliced into 24‐h periods. Data were separated into Excel spreadsheets with the data for each day. For each task, the dependent variables that were analyzed are described below. For readability, IntelliCage statistical output is in Table 1.

**TABLE 1:** IntelliCage significant results.

Adaptations														
Free adaptation														
Dependent variable	ME geno		ME sex		ME day		Geno × Sex		Geno × Day		Sex × Day		Geno × Sex × Day	
	*F*	*p*	*F*	*p*	*F*	*p*	*F*	*p*	*F*	*p*	*F*	*p*	*F*	*p*
Total visits					*F*_(2,82)_ = 11.253	*p* < 0.001								
Total licks					*F*_(2,82)_ = 16.045	*p* < 0.001								

Door adaptation														
Dependent variable	ME geno		ME sex		ME day		Geno × Sex		Geno × Day		Sex × Day		Geno × Sex × Day	
	*F*	*p*	*F*	*p*	*F*	*p*	*F*	*p*	*F*	*p*	*F*	*p*	*F*	*p*
Total visits					*F*_(1,40)_ = 5.654	*p* = 0.022								
Total licks					*F*_(1,40)_ = 10.912	*p* = 0.002								

Nose poke adaptation														
Dependent variable	ME geno		ME sex		ME day		Geno × Sex		Geno × Day		Sex × Day		Geno × Sex × Day	
	*F*	*p*	*F*	*p*	*F*	*p*	*F*	*p*	*F*	*p*	*F*	*p*	*F*	*p*
Total visits			*F*_(1,40)_ = 9.496	*p* = 0.004	*F*_(1,40)_ = 5.352	*p* = 0.026								
Total licks											*F*_(1,40)_ = 9.393	*p* = 0.004		
Visits with a nose poke			*F*_(1,40)_ = 12.542	*p* = 0.001	*F*_(1,40)_ = 5.007	*p* = 0.031								
Visits with ≥ 1 lick			*F*_(1,40)_ = 16.515	*p* < 0.001										

Water restriction adaptation						
Dependent variable	ME geno		ME sex		Geno × Sex	
	*F*	*p*	*F*	*p*	*F*	*p*
Total visits			*F*_(1,42)_ = 4.129	*p* = 0.0485		
Total licks			*F*_(1,42)_ = 7.405	*p* = 0.0094		
Visits with ≥ 1 Lick			*F*_(1,42)_ = 19.17	*p* < 0.0001		
Visits during water access			*F*_(1,42)_ = 12.30	*p* = 0.0011		

Place preference and reversal														
Place preference														
Dependent variable	ME geno		ME sex		ME day		Geno × Sex		Geno × Day		Sex × Day		Geno × Sex × Day	
	*F*	*p*	*F*	*p*	*F*	*p*	*F*	*p*	*F*	*p*	*F*	*p*	*F*	*p*
Total visits			*F*_(1,33)_ = 6.750	*p* = 0.014	*F*_(5,165)_ = 4.127	*p* = 0.001							*F*_(5,165)_ = 4.776	*p* < 0.001
Assigned visits with ≥ 1 Lick			*F*_(1,33)_ = 34.833	*p* < 0.001	*F*_(5,165)_ = 5.557	*p* < 0.001								
%Correct responses					*F*_(5,165)_ = 9.421	*p* < 0.001								

Reversal														
Dependent variable	ME geno		ME sex		ME day		Geno × Sex		Geno × Day		Sex × Day		Geno × Sex × Day	
	*F*	*p*	*F*	*p*	*F*	*p*	*F*	*p*	*F*	*p*	*F*	*p*	*F*	*p*
Total visits			*F*_(1,33)_ = 3.944	*p* = 0.055	*F*_(5,165)_ = 29.266	*p* < 0.001	*F*_(1,33)_ = 8.147	*p* = 0.007	*F*_(5,165)_ = 2.756	*p* = 0.020	*F*_(5,165)_ = 4.890	*p* < 0.001	*F*_(5,165)_ = 7.015	*p* < 0.001
Assigned visits with ≥ 1 Lick			*F*_(1,33)_ = 7.354	*p* = 0.011	*F*_(5,165)_ = 4.802	*p* < 0.001					*F*_(5,165)_ = 3.621	*p* < 0.004		
%Correct responses					*F*_(5,165)_ = 4.891	*p* < 0.001					*F*_(5,165)_ = 3.073	*p* = 0.011		

Reaction time tasks														
Reaction time task 2 s pre‐cue with 7 s cue														
Dependent variable	ME geno		ME sex		ME day		Geno × Sex		Geno × Day		Sex × Day		Geno × Sex × Day	
	*F*	*p*	*F*	*p*	*F*	*p*	*F*	*p*	*F*	*p*	*F*	*p*	*F*	*p*
Total initiated trials	*F*_(1,28)_ = 9.199	*p* = 0.005	*F*_(1,28)_ = 14.528	*p* < 0.001										
%Premature response	*F*_(1,28)_ = 4.078	*p* = 0.053			*F*_(4,112)_ = 16.139	*p* < 0.001								
%Correct responses					*F*_(4,112)_ = 49.543	*p* < 0.001			*F*_(4,112)_ = 4.040	*p* = 0.004				

Reaction time task variable 2, 4, or 8 s pre‐cue with 7 s cue														
Dependent variable	ME geno		ME sex		ME day		Geno × Sex		Geno × Day		Sex × Day		Geno × Sex × Day	
	*F*	*p*	*F*	*p*	*F*	*p*	*F*	*p*	*F*	*p*	*F*	*p*	*F*	*p*
Total initiated trials			*F*_(1,26)_ = 13.199	*p* = 0.001			*F*_(4,104)_ = 2.604	*p* = 0.040						
%Premature responses (all trials)					*F*_(4,104)_ = 10.975	*p* < 0.0001								
%Premature responses (trials with 2 s pre‐cue)					*F*_(4,104)_ = 3.206	*p* = 0.016								
%Premature responses (trials with 4 s pre‐cue)					*F*_(4,104)_ = 9.698	*p* < 0.0001								
%Premature responses (trials with 8 s pre‐cue)					*F*_(4,104)_ = 6.275	*p* < 0.0001								
%Correct responses (all trials)	*F*_(1,26)_ = 5.282	*p* = 0.030			*F*_(4,104)_ = 15.275	*p* < 0.0001	*F*_(4,104)_ = 2.930	*p* = 0.024						
%Correct responses (trials with 2 s pre‐cue)					*F*_(4,104)_ = 3.104	*p* = 0.019								
%Correct responses (trials with 4 s pre‐cue)	*F*_(1,26)_ = 4.614	*p* = 0.041			*F*_(4,104)_ = 13.987	*p* < 0.0001								
%Correct responses (trials with 8 s pre‐cue)	*F*_(1,26)_ = 4.614	*p* = 0.041			*F*_(4,104)_ = 9.168	*p* < 0.0001					*F*_(4,104)_ = 3.805	*p* = 0.006		

Reaction time task variable 2, 4, or 8 s pre‐cue with 3 s cue														
Dependent variable	ME geno		ME sex		ME day		Geno × Sex		Geno × Day		Sex × Day		Geno × Sex × Day	
	*F*	*p*	*F*	*p*	*F*	*p*	*F*	*p*	*F*	*p*	*F*	*p*	*F*	*p*
Total initiated trials	*F*_(1,25)_ = 16.518	*p* < 0.0001	*F*_(1,25)_ = 21.992	*p* < 0.0001	*F*_(4,100)_ = 13.044	*p* < 0.0001	*F*_(1,25)_ = 5.124	*p* = 0.033	*F*_(4,100)_ = 7.874	*p* < 0.0001	*F*_(4,100)_ = 2.737	*p* = 0.033	*F*_(4,100)_ = 5.590	*p* < 0.0001
%Premature responses (all trials)	*F*_(1,25)_ = 4.790	*p* = 0.038			*F*_(4,100)_ = 3.330	*p* = 0.013								
%Premature responses (trials with 2 s pre‐cue)									*F*_(4,100)_ = 3.347	*p* = 0.013				
%Premature responses (trials with 4 s pre‐cue)	*F*_(1,25)_ = 4.667	*p* = 0.041												
%Premature responses (trials with 8 s pre‐cue)	*F*_(1,25)_ = 5.473	*p* = 0.028												
%Correct responses (all trials)					*F*_(4,100)_ = 2.966	*p* = 0.023								
%Correct responses (trials with 2 s pre‐cue)									*F*_(4,100)_ = 2.849	*p* = 0.028				
%Correct responses (trials with 4 s pre‐cue)	*F*_(1,25)_ = 4.383	*p* = 0.047												
%Correct responses (trials with 8 s pre‐cue)	*F*_(1,25)_ = 7.509	*p* = 0.011			*F*_(4,104)_ = 2.938	*p* = 0.024								

Reaction time task variable 2, 4, or 8 s pre‐cue with 1 s cue														
Dependent variable	ME geno		ME sex		ME day		Geno × Sex		Geno × Day		Sex × Day		Geno × Sex × Day	
	*F*	*p*	*F*	*p*	*F*	*p*	*F*	*p*	*F*	*p*	*F*	*p*	*F*	*p*
Total initiated trials	*F*_(1,25)_ = 5.681	*p* = 0.025	*F*_(1,25)_ = 5.534	*p* = 0.027										
%Premature responses (all trials)													*F*_(4,100)_ = 3.182	*p* = 0.017
%Premature responses (trials with 2 s pre‐cue)														
%Premature responses (trials with 4 s pre‐cue)														
%Premature responses (trials with 8 s pre‐cue)									*F*_(4,100)_ = 2.656	*p* = 0.037			*F*_(4,100)_ = 2.656	*p* = 0.037
%Correct responses (all trials)			*F*_(1,25)_ = 4.497	*p* = 0.044	*F*_(4,100)_ = 4.697	*p* = 0.002								
%Correct responses (trials with 2 s pre‐cue)					*F*_(4,100)_ = 2.705	*p* = 0.035								
%Correct responses (trials with 4 s pre‐cue)														
%Correct responses (trials with 8 s pre‐cue)			*F*_(1,25)_ = 5.321	*p* = 0.030										

Place avoidance, and retention and extinction						
Place avoidance						
Dependent variable	ME geno		ME sex		Geno × Sex	
	*F*	*p*	*F*	*p*	*F*	*p*
Total visits			*F*_(1,25)_ = 10.25	*p* = 0.0037		
Assigned visits			*F*_(1,25)_ = 6.912	*p* = 0.0144	*F*_(1,25)_ = 6.664	*p* = 0.0161

Retention and extinction														
Dependent variable	ME geno		ME sex		ME day		Geno × Sex		Geno × Day		Sex × Day		Geno × Sex × Day	
	*F*	*p*	*F*	*p*	*F*	*p*	*F*	*p*	*F*	*p*	*F*	*p*	*F*	*p*
Total visits			*F*_(1,25)_ = 5.380	*p* = 0.029										
Total licks					*F*_(1,50)_ = 12.425	*p* < 0.0001			*F*_(1,50)_ = 13.117	*p* < 0.0001				
Assigned visits with nosepoke	*F*_(1,25)_ = 5.067	*p* = 0.033	*F*_(1,25)_ = 78.443	*p* < 0.0001										

##### 2.4.3.1 Adaptation Phases

During the adaptation phases, mice were acclimated to the IntelliCage environment and underwent multiple shaping tasks, described below, to train them on the basic procedure to access water. By the end of adaptation, mice were able to nose poke at a corner to open the doors during the restricted water access period, which allowed water acquisition.

###### 2.4.3.1.1 Free Adaptation (Days 1–3)

During free adaptation, mice were allowed to freely explore and adapt to the new environment. During this phase, all doors were open and all water bottles were accessible. No water restriction was implemented during this session.

The following measures were analyzed for the free adaptation phases:

- Total visits = the total number of all corner visits. This is a measure of exploratory and water‐seeking behavior.
- Total licks = the total number of licks made on a given day. This is a measure of water consumption.

###### 2.4.3.1.2 Door Adaptation (Days 4–5)

During door adaptation, the doors to the water bottles were closed but would open for any corner visit. No water restriction was implemented during this phase. Total visits and total licks were analyzed.

###### 2.4.3.1.3 Nose Poke Adaptation (Days 6–7)

During nose poke adaptation, mice were trained to nose poke to open the doors and access the water. No water restriction period was implemented. In addition to total visits and total licks, the following outcomes for this task were as follows:

- Total visits with nose poke = number of visits with one or more nose pokes, which indicated successful shaping to the task.
- Visits with ≥ 1 lick = number of visits with one or more licks, which indicates the number of visits with clear water‐seeking behavior.

###### 2.4.3.1.4 Water Deprivation Adaptation (Day 8)

In our previous reports using only females, 3xTg‐AD mice displayed more water‐seeking behavior than NonTg mice (Winslow et al. 2021; Judd et al. 2024), likely resulting from demand differences relating to body weight (Bachmanov et al. 2002). To equalize water‐seeking motivation between genotypes, a water restriction protocol was implemented, consistent with reports from others using this apparatus (Masuda et al. 2018). Starting with this adaptation phase, water was only available between 21:00 and 0:00, during the dark phase of the light–dark cycle when mice are active. In addition to the metrics analyzed in nose poke adaptation, the following metric was also assessed:

- Total visits during water access = number of visits made during the 3 h specifically when water could be accessed.

#### 2.4.4 Place Preference (Days 9–14) and Reversal (Days 15–20)

During place preference (PP), each mouse could only access water from one corner (the assigned correct corner). To eliminate corner bias, the correct corner for each mouse during place preference was assigned based on the least‐visited corner during adaptation. During reversal, water was available only from the corner opposite from the correct corner in PP. The assigned corner in reversal was maintained as the correct corner during all subsequent tasks. To prevent overcrowding and learning by imitation, the assigned corners were balanced for genotype and the number of mice assigned to a corner was limited to four, as previously described (Winslow et al. 2021; Mifflin et al. 2021; Judd et al. 2024; Tallino et al. 2024). In addition to measuring total visits and total licks per day, outcomes for this task included:

- Assigned visits with ≥ 1 lick = number of visits to the assigned corner that included at least one lick.
- %Correct responses = (assigned visits with ≥ 1 lick/total visits) × 100

#### 2.4.5 Reaction Time Testing (Days 21–40)

During the reaction time (RT) tasks, when a mouse entered the assigned corner, the first nose poke of a visit initiated a trial. During a pre‐cue delay, mice had to learn to wait for a green LED to illuminate before proceeding, assessing impulsivity and attention. Once the green LED was on, mice could nose poke again, which would open the door and permit water access. Any nose poke before or after the green LED was illuminated resulted in an error and the mouse had to leave the corner, re‐enter, and initiate a new trial to attempt to access water. By manipulating the pre‐cue delay and the time of cue onset, RT tasks progressively increased in difficulty and attentional demand. The RT sessions had the following permutations:

- Days 21–25: A 2‐s pre‐cue followed by 7‐s with the green LED on
- Days 26–30: A variable 2, 4, or 8‐s pre‐cue followed by 7‐s with the green LED on.
- Days 31–35: A variable 2, 4, or 8‐s pre‐cue followed by 3‐s with the green LED on.
- Days 36–40: A variable 2, 4, or 8‐s pre‐cue followed by 1‐s with the green LED on.

We measured the following outcomes for the initiated trials:

- Trials = the number of initiated trials.
- %Premature responses = (total trials with premature response/total trials) × 100. A response was premature when a nose poke occurred during the pre‐cue delay period. This is a measure of impulsivity.
- %Correct responses = (total trials with correct response/total trials) × 100. A response was correct when a nose poke occurred during the time the green LED cue was on.

Additionally, for the sessions with a variable pre‐cue duration, these metrics were broken down by the individual pre‐cue durations (ex. %Premature responses with 2‐s pre‐cue = (premature responses with 2‐s pre‐cue/trials with 2‐s pre‐cue) × 100).

#### 2.4.6 Avoidance and Extinction (Days 41–46)

Place avoidance tasks tap into hippocampal and amygdalar function (Oler et al. 2005) and included both training (learning) and probe (memory and extinction) trials. For the 1‐day training trial (avoidance training), a nose poke in the reward corner resulted in an air puff (~0.8 bar, 1‐s air puff). The doors in all corners remained closed and water was not available during the avoidance phase. In addition to measuring total visits, we also analyzed the number of corner visits with nose pokes at the air puff corner to assess working memory errors.

After avoidance training, the mice were transferred to standard home cages for a 48‐h delay with water *ad libitum*. After the delay, the mice were returned to the IntelliCage for 3 days with water available at all four corners and the air puff stimulus removed to assess memory retention and extinction.

### 2.5 Blood Collection and Plasma Extraction

Blood (≤ 1% of the subject's body weight) was collected via the submandibular vein and then placed into K_2_ EDTA‐lined tubes and then inverted 10 times to ensure anticoagulation. About 250–300 μL of blood was collected at euthanasia. Tubes were kept on ice for 60 min and centrifuged at 455 × *g* for 30 min at 4°C. The top (plasma) layer was collected and stored at −80°C. Plasma was collected at months 3, 6, 9, 12, and at euthanasia.

### 2.6 Tissue Harvesting and Processing

Mice were euthanized at an average 16.81 months of age. The age range of mice at euthanasia was 15.37–18.03 months of age. All mice were perfused with 1× PBS, and the hippocampus (HP), cortex (CTX), and liver were dissected and flash‐frozen on dry ice. This tissue was homogenized in tissue protein extraction reagent (T‐PER) supplemented with protease and phosphatase inhibitors and prepared for protein assays as previously described (Winslow et al. 2021; Judd et al. 2023; Dave et al. 2021, 2023).

### 2.7 ELISA and Choline Assays

Commercially available ELISA kits (Invitrogen‐ThermoFisher Scientific) were used to measure levels of insoluble Aβ40 (KHB3481) and Aβ42 (KHB3441), as well as phosphorylated tau (pTau) at Threonine (T)181 (KHO0631) and Serine (S)396 (KHB7031), as previously described (Velazquez et al. 2019; Judd et al. 2023; Dave et al. 2021, 2023). Briefly, tissue homogenates were placed in 96‐well plates for processing and in precoated, flat‐bottom 96‐well plates for reading in a plate reader (BioTek) at 450 nm, based on the manufacturer's instructions. To quantify choline levels, commercially available kits (Abcam, ab219944) were used. Plasma samples were placed in 96‐well plates with reagents and read on a plate reader (BioTek) at 590 nm, as directed by the manufacturer's instructions.

### 2.8 Western Blots

Liver homogenates und immunoblot analysis to quantify PEMT which is abundantly expressed in the liver, where endogenous choline biosynthesis occurs (Blusztajn 1998). Immunoblotting was performed under reducing conditions as previously described (Winslow et al. 2021; Velazquez et al. 2017; Judd et al. 2023; Dave et al. 2023). Proteins were resolved on NuPAGE 10% Bis‐Tris Midi Gels (Invitrogen, WG1203BX10). SeeBlue Plus2 Pre‐stained Protein Standard (Invitrogen, LC5925) was used as the molecular weight ladder. Membranes were probed with anti‐PEMT (1:1000; Thermo Fisher Scientific, PA5‐42383, predicted molecular weight between 15 and 20 kDa) and anti‐GAPDH (1:5000; Abcam, ab8245) as a loading control. Band intensities were quantified using LI‐COR Image Studio software. The intensity of each protein of interest was normalized to its corresponding loading control within the same blot. A shared between‐blot control sample was included on every blot to allow normalization across blots and control for inter‐blot variability. All analyses were performed with the experimenter blinded to group allocations.

### 2.9 Statistical Analyses

Unpaired *t*‐tests, two‐way analysis of variance (ANOVA), and Chi‐Squared tests were performed using GraphPad Prism (version 10.1.1). Repeated measures ANOVAs were performed using SPSS (v28.0.1.1). Post hoc analyses were performed with Bonferroni corrections, except for chow consumption analysis, which utilized least squared differences (LSD) post hoc test because the analysis was at the cage level, reducing the *n*'s. Significance was set at *p* < 0.05.

## 3 Results

### 3.1 3xTg‐AD Males Had a High Rate of Mortality Compared to Other Groups

More 3xTg‐AD mice died before the end of the study than NonTg mice (Figure 1C); the survival curve of the groups significantly differed (*X* ^*2*^ (3, *N* = 75) = 15.10, *p* = 0.0017). While 87.5% of NonTg females and 80.0% of NonTg males survived to the end of the study, only 52.2% of 3xTg‐AD females and 20.8% of 3xTg‐AD males survived to the end.

### 3.2 NonTg Males Weighed More Than Their 3xTg‐AD and Female Counterparts Through 12 Months of Age

While mice gained weight through most of the study, all groups started to lose weight after 12 months (Figure 1D). Consequently, body weight was analyzed in two phases.

Weight from 2 to 12 months (Figure 1E), before the start of behavioral testing, was assessed. A genotype by sex interaction (*F*_(1,57)_ = 12.32, *p* = 0.0009) revealed that 3xTg‐AD females gained more weight than their NonTg counterparts (*p* = 0.0282), but 3xTg‐AD males gained less weight than their NonTg counterparts (*p* = 0.0091). Additionally, for NonTg mice, males gained more weight than females (*p* = 0.0014).

Next, we phase‐assessed weight differences from 12 months of age until the end of the study (Figure 1F). Males lost more weight during this phase than females (*F*_(1,36)_ = 12.37, *p* = 0.0012). A genotype by sex interaction (*F*_(1,36)_ = 4.840, *p* = 0.0343) revealed that NonTg males lost more weight than their female counterparts (*p* = 0.0282). At the endpoint measure, the only measure taken following behavioral testing, body weights were similar between all the groups (Figure 1G). This largely reflects a decline in weight in the NonTg males. These results demonstrate that while males, particularly NonTg males, gained more weight than females across the lifespan, weight loss at older ages resulted in similar body weights at the end of the study.

### 3.3 3xTg‐AD Females and NonTg Males Consume More Chow Than Other Groups

Chow consumption data was analyzed at 3, 6, 9, and 12 months of age, where each data point on the graphs for this analysis is an average of food consumption per cage. At 3 months of age (Figure 1H), females consumed less chow than males (*F*_(1,20)_ = 19.07, *p* = 0.0003). Additionally, a genotype by sex interaction (*F*_(1,20)_ = 46.27, *p* < 0.0001) revealed that at 3 months of age, both 3xTg‐AD females and NonTg males consumed more chow than both 3xTg‐AD males (*p* = 0.0435 and *p* < 0.0001, respectively) and NonTg females (*p* = 0.0008 and *p* < 0.0001, respectively). At 6 months of age (Figure 1I), a genotype by sex interaction (*F*_(1,20)_ = 13.22, *p* = 0.0016) showed that 3xTg‐AD females (*p* = 0.0007) and NonTg males (*p* = 0.0046) consumed more chow than NonTg females. At 9 months of age (Figure 1J), a genotype by sex interaction (*F*_(1,18)_ = 6.197, *p* = 0.0228) again showed that NonTg males consumed more than their female counterparts (*p* = 0.0164). At 12 months of age (Figure 1K), a genotype by sex interaction (*F*_(1,18)_ = 13.20, *p* = 0.0019) showed that 3xTg‐AD females consumed more than both 3xTg‐AD males (*p* = 0.0423) and NonTg females (*p* = 0.0015), and that NonTg males consumed more than NonTg females (*p* = 0.0095). Together, this shows that 3xTg‐AD females and NonTg males repeatedly consumed more chow than other groups, reflecting higher body weight gain throughout most of the study.

### 3.4 3xTg‐AD Mice Exhibit Lower Plasma Choline Levels Than NonTg Mice Across the Lifespan

To determine whether circulating choline levels differ across genotype, sex, and age, we analyzed plasma levels at 3, 6, 9, 12 months, and at endpoint (an average of 16.81) (Figure 1L). Choline levels were lower in 3xTg‐AD than in NonTg mice across the lifespan (*F*_(1,35)_ = 56.080, *p* < 0.001). Further, choline levels decreased across the lifespan (*F*_(4,140)_ = 103.709, *p* < 0.001). An age by genotype interaction (*F*_(4,140)_ = 16.465, *p* < 0.001) revealed that this was primarily in the NonTg animals, whose choline levels decreased at each timepoint (*p* < 0.001). However, in 3xTg‐AD mice at the end point measure, choline levels were lower than at 6 (*p* = 0.001), 9 (*p* = 0.02), and 12 (*p* = 0.045) months of age, but no other changes across time were observed. Additionally, an age by sex by genotype interaction (Figure 1M; *F*_(4,140)_ = 4.705, *p* = 0.001) revealed that at 3 months of age, NonTg female mice had higher choline levels than their male counterparts. Together, these data indicate that genotype differences are evident from a young age and that choline levels decline with age in both genotypes, with the greatest age‐related reductions observed in NonTg mice.

### 3.5 PEMT Protein Levels Were Higher in Females but Unchanged Across Genotypes

To determine the levels of the PEMT protein within the liver, the enzyme responsible for endogenous choline production, we performed immunoblot analysis and examined effects across genotype, age, and sex (Figure 1M,N). We found that PEMT protein levels were higher in female compared to male mice (*F*_(1,35)_ = 7.661, *p* = 0.009). Consistent with our previous report, we did not find PEMT differences based on genotype (Dave et al. 2023). Next, we correlated PEMT protein levels with circulating choline levels at 12 months (*r*_(38)_ = 0.2639, *p* = 0.1045) and at end point measure (*r*_(38)_ = 0.2025, *p* = 0.2164) and found no significant differences. This suggests that differences in circulating choline levels may not be the result of endogenous production but may likely reflect an interplay with metabolic demand.

### 3.6 Behavior

#### 3.6.1 3xTg‐AD Mice Explored Less Than NonTg Mice on the Elevated Plus Maze (EPM)

To assess anxiety‐like behavior, we used the EPM and analyzed time in open arms, number of open arm entries, and number of total arm entries. Time in open arms (Figure 1O) was similar across all groups; however, 3xTg‐AD mice made fewer open arm entries (Figure 1P; *F*_(1,45)_ = 7.347, *p* = 0.009) and fewer total arm entries (Figure 1Q; *F*_(1,46)_ = 7.254, *p* = 0.0098) than NonTg. Together this suggests that 3xTg‐AD mice were less exploratory than NonTg and may have reflected higher anxiety‐like behavior, but no sex differences were noted.

#### 3.6.2 3xTg‐AD Performed Better Than NonTg on Day 2 of the Rotarod, but All Groups Had Similar Performance on the Probe Test Day

Rotarod was used to assess motor learning and endurance (Figure 1R). All groups performed similarly on Day 1. On Day 2, 3xTg‐AD mice had a higher latency to fall (stayed on the spinning rod longer) than NonTg (*F*_(1,47)_ = 4.779, *p* = 0.0338). On Day 3, the probe challenge day, all groups had similar performance. Together, this shows that NonTg mice were able to recover from performance issues on Day 2 and group differences were transitory.

#### 3.6.3 IntelliCage

##### 3.6.3.1 During the Adaptation Sessions, All Groups Learned the Rules of the Task, and Males Exhibited Higher Water‐Seeking Behavior Than Females

To learn the rules of the IntelliCage tasks, mice underwent adaption phases (Figure S1A–C). During free adaptation, fewer total visits (Figure S1D) were made on Day 3 than on Days 1 and 2 (*p* < 0.001), but more licks (Figure S1E) were made on Days 2 and 3 (*p* < 0.001) than on Day 1. During door adaptation, total visits (Figure S1F) and licks (Figure S1G) increased from Day 4 to Day 5. During nose poke adaptation (Figure S1H), visits to corners increased from Day 6 to Day 7, and males made more total visits than females. For licks made during nose poke adaptation (Figure S1I), a significant interaction of day by sex revealed that females increased their total licks from Day 6 to Day 7 (Figure S1J; *p* = 0.002) and males trended to make more licks than females on Day 6 (*p* = 0.065). Males made more visits with ≥ 1 lick (Figure S1K) than females. During water restriction adaptation, males made more total visits (Figure S1L), total licks (Figure S1M), visits with ≥ 1 lick (Figure S1N), and more visits during the water access period (Figure S1O) than females. While some sex differences were observed across the adaptation sessions, all groups were able to acquire the basic rules of the task, learning to nose poke to acquire water.

##### 3.6.3.2 Preference for the Correct Corner Increased Across the Days of Place Preference (PP) and Reversal

**FIGURE 2:**
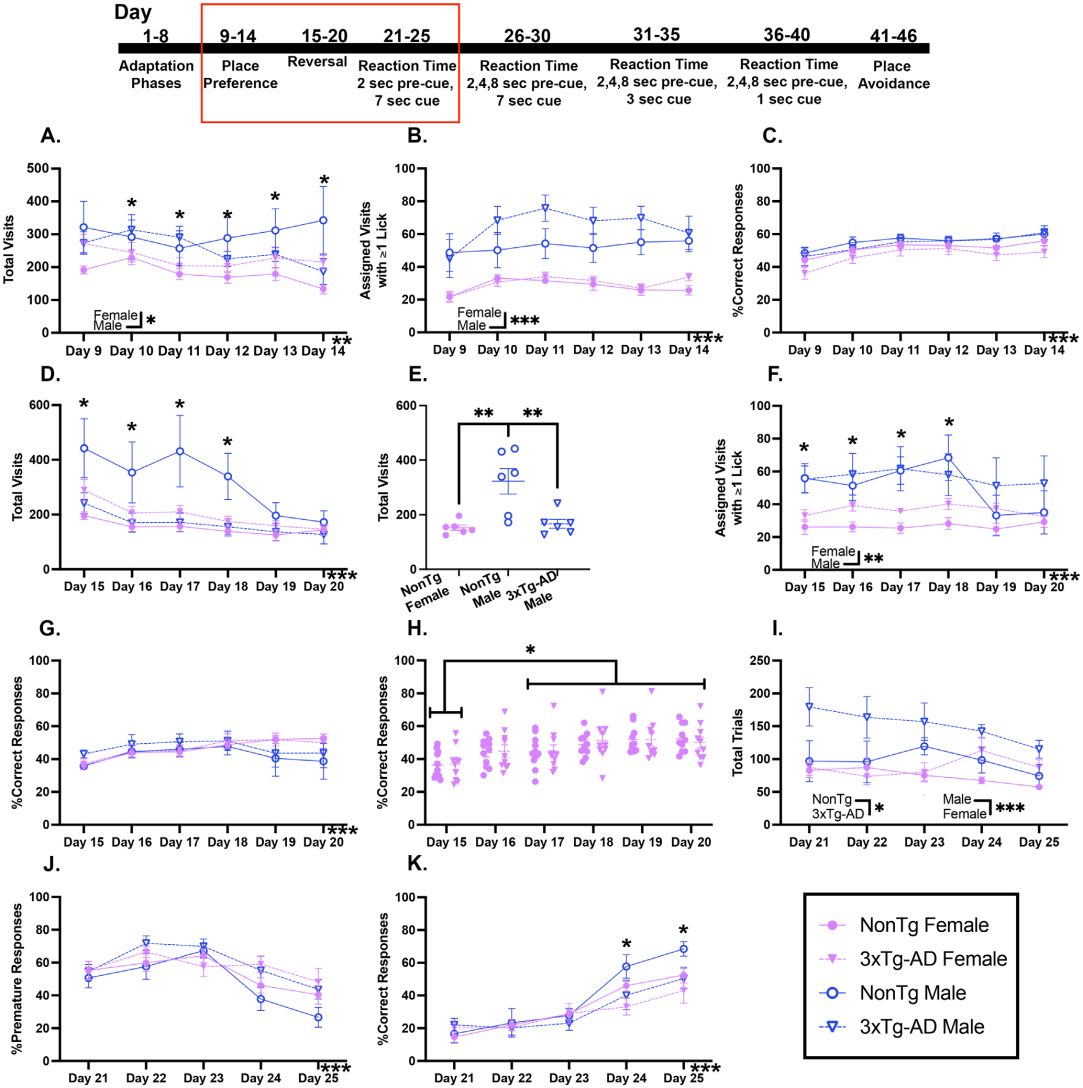
All groups improved in place preference (PP), reversal, and reaction time (RT) tasks with 2‐s pre‐cue and 7‐s cue across days. (A) During PP, males made significantly more total visits than females. (B) Assigned visits with ≥ 1 lick during PP increased across days and males made more assigned visits with ≥ 1 lick than females. (C) The %Correct responses during PP increased across days. (D) For reversal, total visits decreased across days and (E) were lower in 3xTg‐AD males and NonTg females than in NonTg males. (F) Males made more assigned visits with ≥ 1 lick than females. (G) The %Correct responses increased across days and (H) females had more correct responses on Days 17–20 than on Day 15. (I) During the RT task with 2‐s pre‐cue and 7‐s cue, there were more total initiated trials by 3xTg‐AD mice than NonTg and more by males than females. The (J) %Premature responses decreased across days. (K) %Correct responses increased across days and were lower in 3xTg‐AD than NonTg on Days 24 and 25. Data are reported as means ± SEM. *p < 0.05, **p < 0.01, ***p < 0.001, ****p < 0.0001.

During PP, there were significantly fewer total visits (Figure 2A) on Day 10 than on Day 12 (*p* = 0.025). Males made more visits than females. Further, a day by genotype by sex interaction showed that 3xTg‐AD females made fewer total visits than 3xTg‐AD males on Day 11 (*p* = 0.041), but 3xTg‐AD males made fewer visits than NonTg males on Day 14 (*p* = 0.023). Additionally, NonTg males made more total visits than NonTg females on Days 12–14 (*p* ≤ 0.01). Assigned visits with ≥ 1 lick increased across days (Figure 2B), with more on Days 10–12 (*p* < 0.05) than on Day 9. Additionally, males made more assigned visits with ≥ 1 lick than females. The %Correct responses (Figure 2C) increased across days and was significantly higher on Days 10–14 (*p* < 0.05) than on Day 9. Together, this shows that all groups improved across days, but that males had a higher success rate than females.

For reversal, total visits (Figure 2D) decreased across days, with fewer on Days 16–19 than on Day 15 (*p* < 0.05). Males trended to make more total visits than females (*p* = 0.055). Further, an interaction of genotype by sex showed that NonTg males made more total visits (Figure 2E) than 3xTg‐AD males (*p* = 0.007) and NonTg females (*p* = 0.003). Groups also differed in number of visits across days. A day by sex interaction revealed that males made more total visits than females on Days 15, 17, and 18 (*p* < 0.05); a day by genotype interaction showed that fewer total visits were made by 3xTg‐AD mice on Days 17 and 18 (*p* <: 0.05) than by NonTg. Finally, a day by genotype by sex interaction showed that 3xTg‐AD males made fewer total visits than NonTg males on Days 15–18 (*p* < 0.05).

Assigned visits with ≥ 1 lick during reversal decreased across days (Figure 2F), however, more were made on Day 18 than on Days 19 and 20 (*p* < 0.05). Males made more assigned visits with ≥ 1 lick than females overall, and an interaction of day by sex showed that males specifically made more assigned visits with ≥ 1 lick than females on Days 15–18 (*p* < 0.01). The %Correct responses increased across days of reversal (Figure 2G), with more on Days 16–18 (*p* < 0.01) than Day 15. Further, a day by sex interaction (Figure 2H) revealed that females increased their %Correct responses across days; %Correct responses were higher on Days 17–20 (*p* < 0.05) than on Day 15. Taken together, this data highlights that all groups increased the proportion of correct responses across days, and males engaged in more water‐seeking across PP and reversal.

##### 3.6.3.3 3xTg‐AD Mice Were Less Successful Than NonTg in the Reaction Time (RT) Task With 2‐s Pre‐Cue and 7‐s Cue Onset

During the first RT task, more trials were initiated (Figure 2I) by 3xTg‐AD mice than NonTg, and males initiated more trials than females. For all groups, the %Premature responses (Figure 2J) decreased across days; the %Premature responses were lower on Day 25 than on Days 21–24 (*p* < 0.05). The %Premature responses of 3xTg‐AD mice trended to be higher than for NonTg (*p* = 0.056). Conversely, all groups increased their %Correct responses (Figure 2K) across days; the %Correct responses were higher on Day 25 than on Days 21–24 (*p* < 0.05). Additionally, an interaction of day by genotype revealed that the %Correct responses for 3xTg‐AD mice were lower than for NonTg on Days 24 and 25 (*p* < 0.05). Together, this shows that in the first RT task, 3xTg‐AD mice showed signs of impulsivity, with lower %Correct and higher %Premature responses than NonTg mice, but the only sex effect was that males engaged in more water seeking than females.

##### 3.6.3.4 For RT With Variable 2, 4, or 8 s Pre‐Cue and 7 s Cue, %Correct Response Was Lower for 3xTg‐AD Than NonTg Mice

**FIGURE 3:**
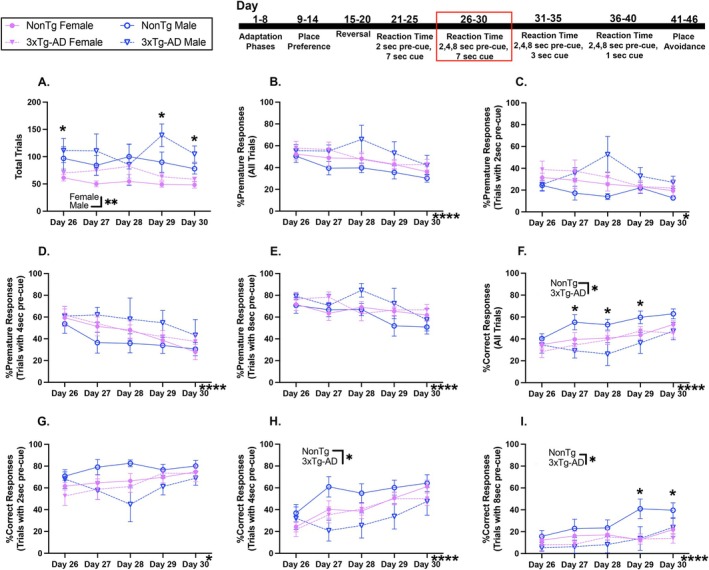
3xTg‐AD mice had lower %Correct responses during RT with variable 2, 4, or 8‐s pre‐cue and 7‐s cue. (A) Males initiated more trials than females. (B–E) %Premature responses for all trials, %Premature responses with 2‐s pre‐cue, %Premature responses with 4‐s pre‐cue, and %Premature responses with 8‐s pre‐cue decreased across days. (F) %Correct responses for all trials increased across days and were lower in 3xTg‐AD mice than NonTg. (G) %Correct responses with 2‐s pre‐cue increased across days. (H, I) %Correct responses with 4 or 8‐s pre‐cue increased across days and were lower in 3xTg‐AD than in NonTg mice. Data are reported as means ± SEM. *p < 0.05, **p < 0.01, ***p < 0.001, ****p < 0.0001.

For the next RT task, variable pre‐cue delays were implemented to further assess impulsivity. We found that males initiated more trials than females (Figure 3A). There was also a day by genotype by sex interaction for total initiated trials, where 3xTg‐AD males initiated more trials than NonTg males on Day 29 (*p* = 0.014) and Day 30 (*p* = 0.045), and NonTg males initiated more trials than their female counterparts on Day 26 (*p* = 0.048), Day 29 (*p* = 0.011), and Day 30 (*p* = 0.008).

Across the days of this session, impulsivity for all trials (Figure 3B) decreased; the %Premature responses on Days 27–30 were lower (*p* < 0.05) than on Day 26. The %Premature responses with 2‐s pre‐cue decreased across days (Figure 3C), and were lower on Day 30 than on Day 28 (*p* = 0.030). There was also a decrease across days for the %Premature responses with 4‐s pre‐cue (Figure 3D); the %Premature responses on Days 29 and 30 (*p* < 0.01) were lower than on Day 26, and lower on Day 30 than on Day 27 (*p* = 0.037). Likewise, the %Premature responses with 8‐s pre‐cue also decreased across days (Figure 3E); the %Premature responses were lower on Day 30 than on Days 26 and 28 (*p* < 0.01).

The %Correct responses increased across days across all trials, regardless of pre‐cue duration. For the %Correct responses for all trials (Figure 3F), a significant day effect revealed better performance on Day 30 than on Days 26–29 (*p* < 0.01). NonTg mice had more success than 3xTg‐AD for all trials. There was also a significant day by genotype by sex interaction for %Correct responses for all trials, where NonTg males had better performance than 3xTg‐AD males on Days 27–29 (*p* < 0.05), and the %Correct responses were higher for NonTg males than for their female counterparts on Day 29 (*p* = 0.032). Analysis of %Correct responses following a 2‐s pre‐cue (Figure 3G) revealed no significant differences. The %Correct responses with a 4‐s pre‐cue (Figure 3H) increased across days; there was better performance on Day 30 than on Days 26–29 (*p* < 0.05). There was also a significant effect of genotype, where NonTg mice had a higher %Correct response than 3xTg‐AD. The %Correct responses with an 8‐s pre‐cue (Figure 3I) also increased across days; there was a higher %Correct response on Day 30 than on Days 26–29 (*p* < 0.05). NonTg mice performed better than 3xTg‐AD. A significant day by sex interaction for %Correct response with an 8‐s pre‐cue revealed that the %Correct responses were higher in males than in females on Days 29 and 30 (*p* = 0.025). In summary, for all groups, the %Premature responses decreased while the %Correct responses increased across days, illustrating that all groups were able to improve on this task, and notably 3xTg‐AD mice had a lower %Correct response than NonTg. The lower %Correct response in trials with higher pre‐cue delay periods illustrates the increase difficulty and demand of this task.

##### 3.6.3.5 For RT With Variable 2, 4, 8 s Pre‐Cue and 3 s Cue, 3xTg‐AD Mice Had Higher %Premature Responses and Lower %Correct Responses Than NonTg

**FIGURE 4:**
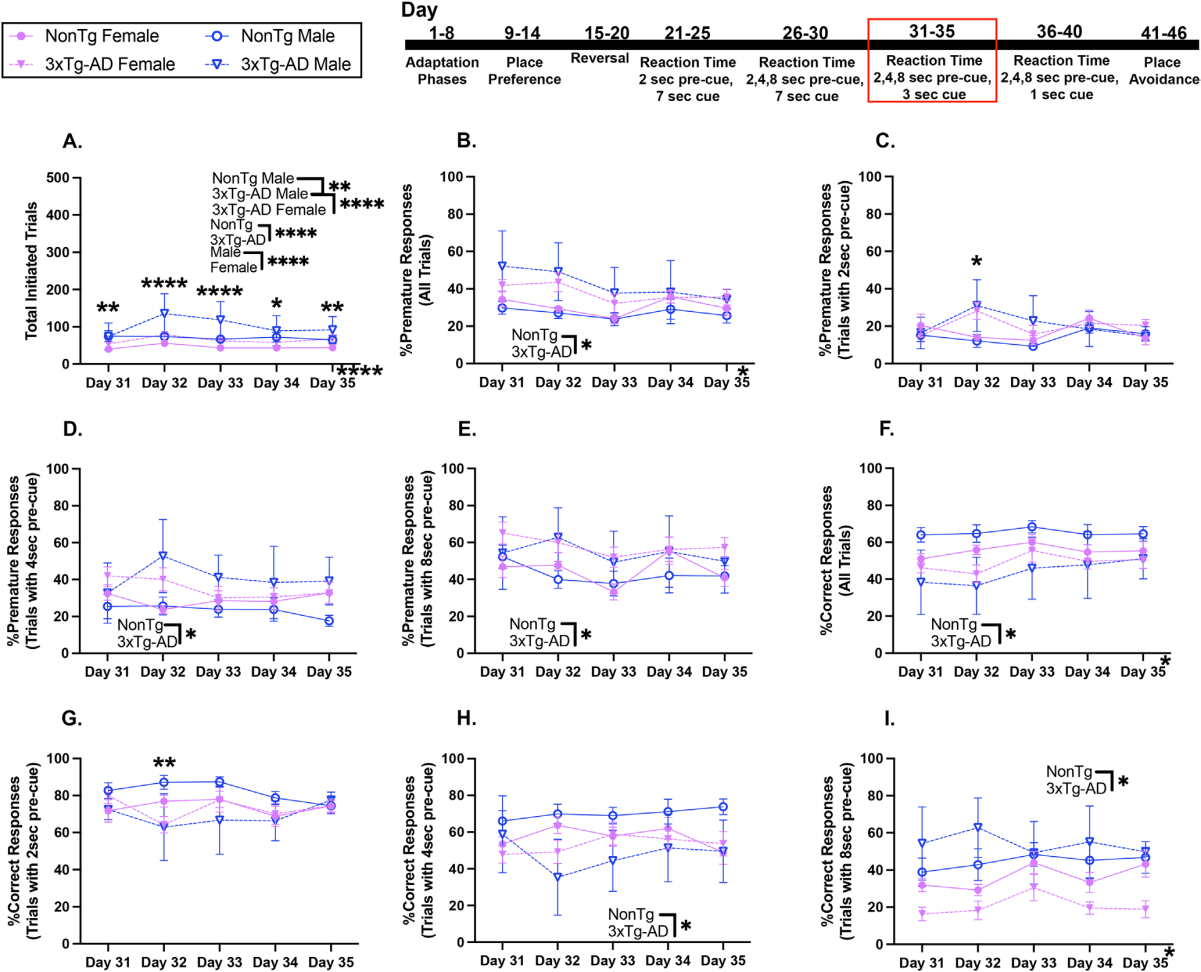
3xTg‐AD mice had lower %Correct responses during RT with variable 2, 4, or 8‐s pre‐cue and 3‐s cue. (A) 3xTg‐AD mice initiated more trials than NonTg, and males initiated more trials than females. (B) %Premature responses for all trials decreased across days and were higher in 3xTg‐AD mice than NonTg. (C) %Premature response with a 2‐s pre‐cue was higher in 3xTg‐AD mice than NonTg on Day 32. (D) %Premature responses with 4‐s pre‐cue and (E) %Premature response with 8‐s pre‐cue were higher in 3xTg‐AD mice than NonTg. (F) %Correct responses for all trials increased across days and were lower in 3xTg‐AD mice than in NonTg. (G) %Correct responses with 2‐s pre‐cue were lower in 3xTg‐AD mice than in NonTg on Day 32. (H, I) %Correct responses with 4 or 8‐s pre‐cue were lower in 3xTg‐AD mice than in NonTg. Data are reported as means ± SEM. *p < 0.05, **p < 0.01, ***p < 0.001, ****p < 0.0001.

In this RT task, pre‐cue delays remained variable and cue onset was shortened to 3 s. For total initiated trials (Figure 4A), 3xTg‐AD mice initiated more trials than NonTg, and males initiated more trials than females. A genotype by sex interaction showed that 3xTg‐AD males initiated more trials than NonTg males (*p* = 0.001) and 3xTg‐AD females (*p* < 0.0001), but NonTg males initiated more trials than their female counterparts (*p* = 0.022).

For %Premature responses for all pre‐cue type trials (Figure 4B), there was a significant effect of day, where a higher %Premature response was found on Day 32 than Day 33 (*p* = 0.005). Additionally, 3xTg‐AD mice had higher %Premature responses for all trials than NonTg. For %Premature response with a 2 s pre‐cue (Figure 4C), there was a significant day by genotype interaction, where %Premature responses were higher for 3xTg‐AD mice than NonTg on Day 32 (*p* = 0.003). For both %Premature responses with 4‐s pre‐cue (Figure 4D) and 8‐s pre‐cue delay (Figure 4E), 3xTg‐AD showed more impulsivity than NonTg.

The %Correct responses for all trials increased across days (Figure 4F), with better performance on Day 33 than on Day 32 (*p* = 0.009). The %Correct responses for all trials were lower in 3xTg‐AD mice than in NonTg. For the %Correct responses with 2‐s pre‐cue delay (Figure 4G), there was a significant day by genotype interaction, where the %Correct responses on Day 32 were lower in 3xTg‐AD mice than in NonTg (*p* = 0.005). The %Correct responses with 4‐s pre‐cue were lower in 3xTg‐AD mice than in NonTg (Figure 4H). For %Correct responses with 8‐s pre‐cue, there was a significant increase with day (Figure 4I), and better performance on Day 33 than on Day 32 (*p* = 0.014). Additionally, the %Correct responses were lower in 3xTg‐AD mice on trials with an 8‐s pre‐cue than in NonTg. Taken together, while all groups improved during this RT task, with %Correct increasing across days, 3xTg‐AD mice showed greater impulsivity than NonTg as evident by higher %Premature response.

##### 3.6.3.6 For RT With Variable 2, 4, or 8‐s Pre Cue and 1‐s Cue, %Correct Responses Were Higher in Males Than in Females

**FIGURE 5:**
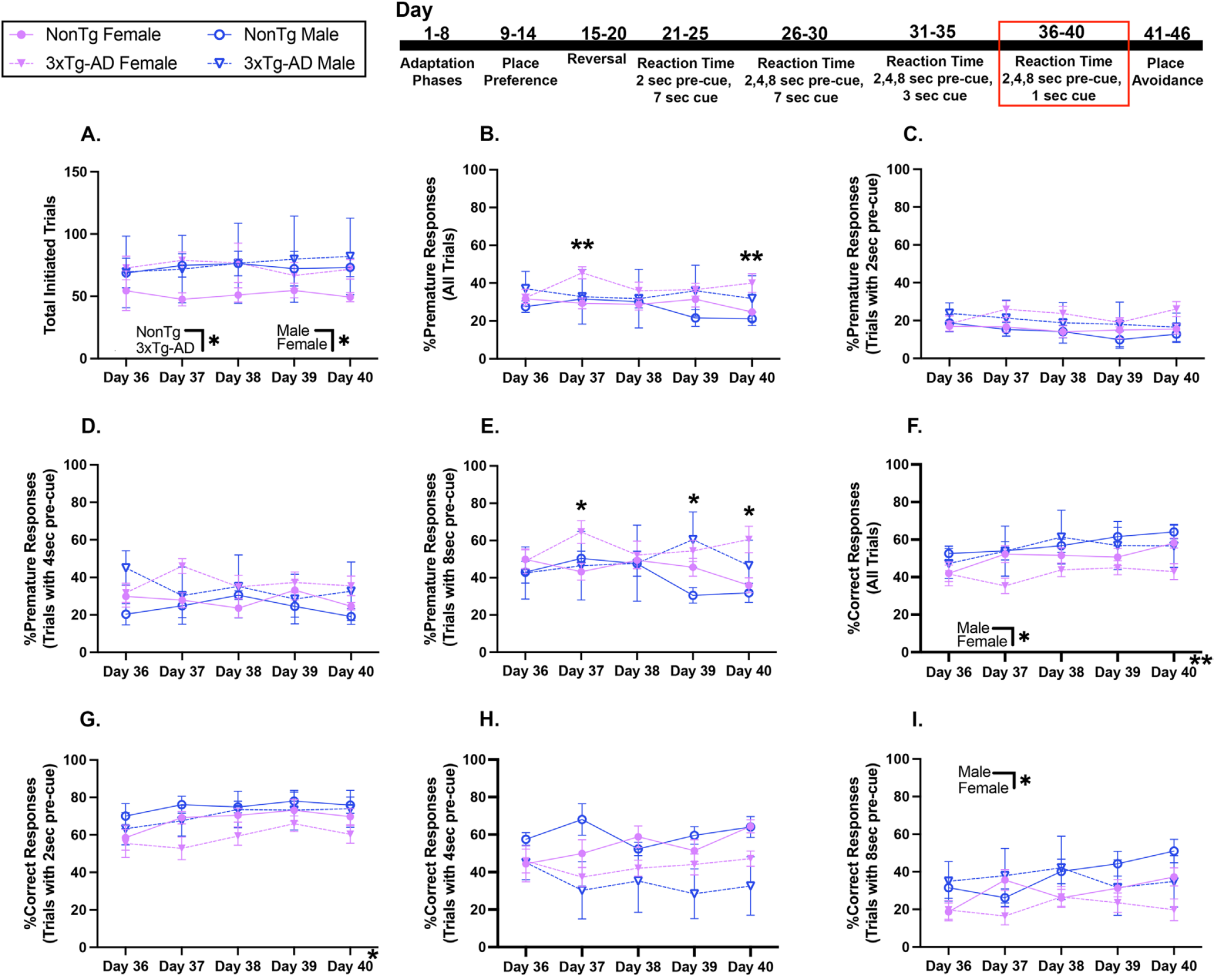
In RT with variables 2, 4, or 8‐s pre‐cue and 1‐s cue, males had a higher %Correct responses than females. (A) 3xTg‐AD mice initiated more trials than NonTg, and males initiated more than females. (B) %Premature responses for all pre‐cue durations were higher in 3xTg‐AD females than NonTg females on Days 37 and 40. There were no significant differences for (C) %Premature responses with 2‐s pre‐cue or (D) %Premature responses with 4‐s pre‐cue. (E) %Premature responses with 8‐s pre‐cue were higher in 3xTg‐AD mice than in NonTg on Days 37 and 40, higher in 3xTg‐AD males than NonTg males on Day 39, and higher in 3xTg‐AD females than NonTg females on Days 37 and 40. (F) %Correct Responses for all pre‐cue durations was higher in males than in females. (G) %Correct responses with 2 s pre‐cue had a significant effect of day. There were no significant differences in (H) %Correct responses with 4 s pre‐cue. (I) The %Correct responses with 8 s pre‐cue were higher in males than in females. Data are reported as means ± SEM. *p < 0.05, **p < 0.01, ***p < 0.001, ****p < 0.0001.

In this final RT task, pre‐cue delays remained variable and cue onset was shortened to 1 s. 3xTg‐AD mice initiated more trials than NonTg (Figure 5A), and males initiated more trials than females.

For %Premature responses for all trials (Figure 5B), there was a significant genotype by sex interaction, where 3xTg‐AD females had higher than NonTg females on Day 37 (*p* = 0.004) and 40 (*p* = 0.009). The %Premature responses for trials with 2‐ or 4‐s pre‐cue delays were similar across days and between groups (Figure 5C,D). For %Premature responses with 8‐s pre‐cue (Figure 5E), a significant day by genotype interaction showed higher responses displayed by 3xTg‐AD mice than NonTg on Days 39 and 40 (*p* < 0.05). Additionally, a day by genotype by sex interaction revealed that the %Premature responses by 3xTg‐AD males on trials with 8‐s pre‐cue on Day 39 were higher (*p* = 0.011) than for NonTg males, and that the %Premature responses by 3xTg‐AD females were higher on Days 37 and 40 (*p* < 0.05) than for NonTg females.

For %Correct responses across all trials (Figure 5F), males showed better performance than females. For %Correct responses on trials with a 2‐ or 4‐s pre‐cue delay (Figure 5G,H), performance was similar across days and groups; however, males had higher %Correct responses with an 8 s pre‐cue than females (Figure 5I). Collectively, in the final phase of RT testing, sex differences were observed, where females performed significantly worse than males, and 3xTg‐AD mice continued to show heightened impulsivity, as evident by higher %Premature responses than NonTg.

##### 3.6.3.7 All Groups Learned to Avoid the Aversive Corner but Quickly Extinguished Their Avoidance Behavior During Extinction Sessions; Males Continued to Exhibit Higher Water‐Seeking Behavior

**FIGURE 6:**
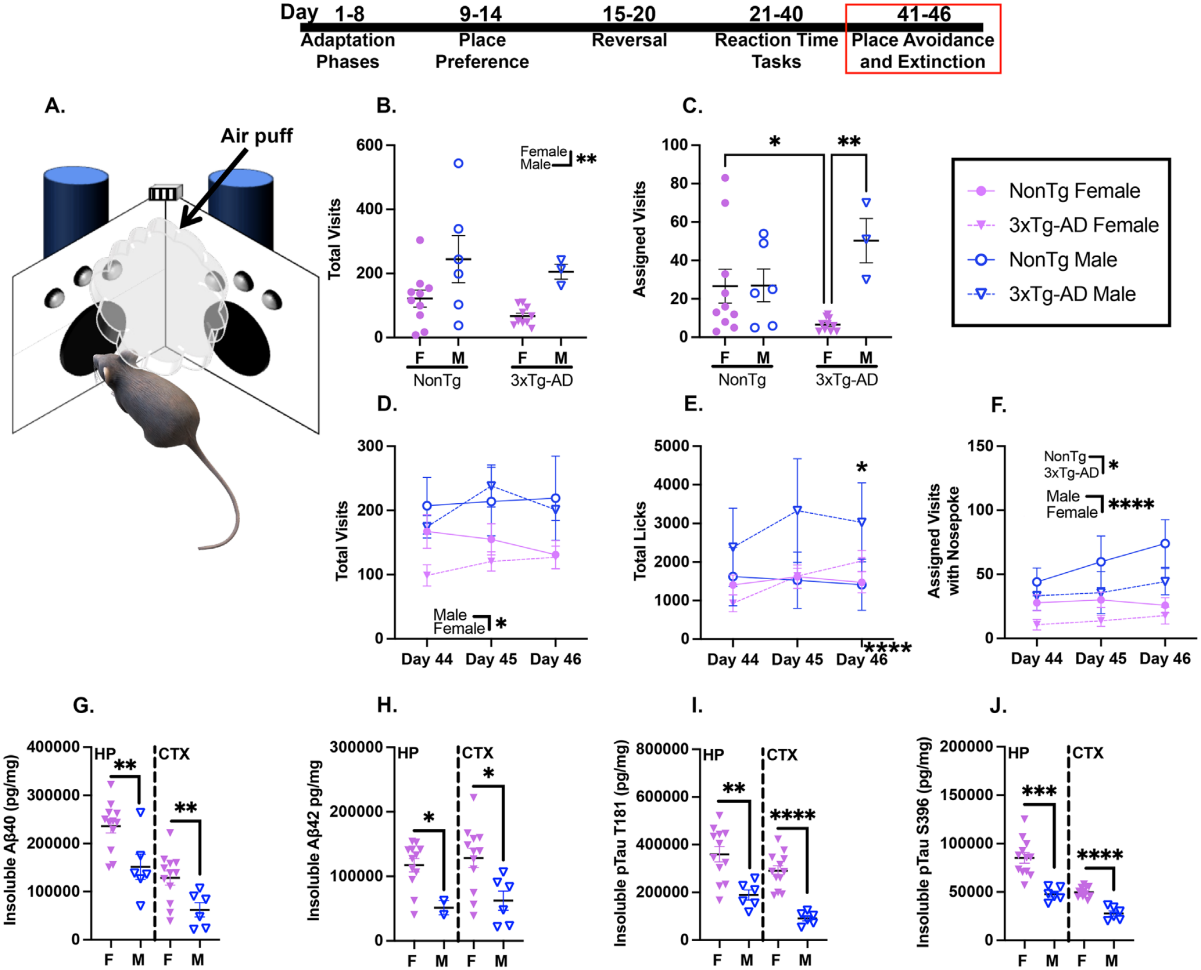
All groups learned to avoid the corners during avoidance and 3xTg‐AD females had higher levels of pathological markers than 3xTg‐AD males. (A) An illustration of an IntelliCage corner delivering an airpuff. During avoidance, males made more (B) total visits and (C) assigned visits than females. During the extinction session days, males made more (D) total visits than females. (E) Total licks increased across days and 3xTg‐AD mice made more licks than NonTg mice on Day 46. Males made more (F) assigned visits with a nose poke than females and NonTg mice made more than 3xTg‐AD mice. Females had higher levels of insoluble (G) Aβ40 and (H) Aβ42 in the hippocampus (HP) and cortex (CTX) than males. For tau phosphorylation at (I) Threonine (T)181, females had higher insoluble levels in the HP and CTX than males. For tau phosphorylation at (J) Serine (S)396, females had higher levels than males in both the HP and CTX. Data are reported as means ± SEM. *p < 0.05, **p < 0.01, ***p < 0.001, ****p < 0.0001.

During the avoidance phase, a nose poke in the assigned corner resulted in an aversive air puff. Water was not available at any corner (Figure 6A). We found that males made more total visits (Figure 6B; *p* = 0.0037) and more visits to the air‐puff corner (Figure 6C; *p* = 0.0144) than females. Further, 3xTg‐AD females made fewer visits to the air‐puff corner than both NonTg females (*p* = 0.0350) and 3xTg‐AD males (*p* = 0.0028).

Following avoidance, mice were placed in standard housing with ad libitum access to chow and water. After 2 days, they were returned to the IntelliCage and underwent memory and extinction sessions. During these sessions, males made more total visits (Figure 6D) than females. Licks (Figure 6E) increased across days, with more total licks on Days 45–46 (*p* < 0.01) than on Day 44. A day by genotype interaction revealed that on Day 46, there were more total licks by 3xTg‐AD mice than NonTg (*p* = 0.032). To assess extinction for avoidance at the assigned corner where a nose poke had led to an air puff during avoidance, assigned visits with a nose poke were assessed (Figure 6F). Males made more assigned visits with a nose poke than females, and NonTg mice made more than 3xTg‐AD.

In summary, males persisted in attempting to access water more during avoidance and more readily returned to water‐seeking during the extinction session than females.

#### 3.6.4 3xTg‐AD Females Display Higher Levels of Amyloidosis and Hyperphosphorylated Tau Than Male Counterparts

To assess sex pathological burdens between male and female 3xTg‐AD mice, we performed ELISAs for insoluble Aβ and tau pathological markers. NonTg brains were not analyzed because they do not have the genes to recapitulate humanized AD pathology (Winslow et al. 2021; Velazquez et al. 2017; Judd et al. 2023; Dave et al. 2023). We found significant sex differences in pathological burden for 3xTg‐AD mice, as anticipated given the pathological inconsistencies between male and female 3xTg‐AD mice (Dennison et al. 2021; Dave et al. 2023; Judd et al. 2023; Velazquez et al. 2017; Winslow et al. 2021). Females had higher levels of insoluble Aβ40 in the hippocampus (HP; Figure 6G left; *t*_(16)_ = 3.076, *p* = 0.0072) and cortex (CTX; Figure 6G right; *t*_(15)_ = 3.926, *p* = 0.0013). Females also had higher levels of insoluble Aβ42 in the HP (Figure 6H left; *t*_(12)_ = 2.481, *p* = 0.0289) and CTX (Figure 6H right; *t*_(16)_ = 2.797, *p* = 0.0129). We also assessed pTau at Threonine (T)181 and Serine (S)396, two pathological tau epitopes. For T181, females had higher insoluble levels in the HP (Figure 6I left; *t*_(16)_ = 3.538, *p* = 0.0027) and CTX (Figure 6I right; *t*_(16)_ = 6.243, *p* < 0.0001). S396 was also higher in females in both the HP (Figure 6J left; *t*_(16)_ = 4.595, *p* = 0.0003) and CTX (Figure 6J right; *t*_(16)_ = 7.711, *p* < 0.0001). Together, these results replicate sexual dimorphism findings in pathological markers in 3xTg‐AD mice (Barber et al. 2024).

## 4 Discussion

The purpose of this study was to determine whether differences in circulating choline levels across the lifespan are associated with sex‐related neuropathological discrepancies observed in the 3xTg‐AD mouse model. 3xTg‐AD mice showed significantly lower plasma choline levels than NonTg mice across the lifespan, while choline levels in NonTg mice declined with age. Notably, differences in choline levels were not readily explainable by differences in food intake or hepatic PEMT expression. Previous work has shown that male 3xTg‐AD mice do not consistently develop the hallmark AD neuropathologies including amyloidosis and hyperphosphorylated tau (Dave et al. 2023; Winslow et al. 2021; Dennison et al. 2021; Barber et al. 2024). Consistent with these previous observations, in the present study, 3xTg‐AD males had lower levels of Aβ and hyperphosphorylated tau markers than 3xTg‐AD females. However, 3xTg‐AD males had lower survival rates than their female counterparts, suggesting a higher impact of peripheral manifestations of disease, potentially driven by the ubiquitously expressed PSEN1 knock‐in mutation. Together, these results expand on known sexual dimorphism in 3xTg‐AD by characterizing choline across the lifespan and differences in endogenous and exogenous choline acquisition.

Genotype differences in plasma choline were observed across the lifespan, both prior to widespread pathological development and continuing into advanced age. While one explanation may be differences due to chow intake, given that NonTg males consumed more compared to other groups, the 3xTg‐AD females also consumed more chow than other groups but had lower plasma choline levels than NonTg females. Another possibility is differences in PEMT activity. However, while sex differences in PEMT were observed, consistent with previous reports that show estrogen actives PEMT and females are more dependent on endogenous production than males (Resseguie et al. 2007), no genotype differences were observed, indicating that choline level genotype differences cannot be explained by PEMT levels. This indicates that intake from chow and PEMT levels are not the only contributors to low choline levels in these mice, but that group differences in metabolic demand or choline absorption could also influence levels. Further, given that 3xTg‐AD females consumed more chow than 3xTg‐AD males at multiple time points, differences in choline intake and PEMT levels cannot explain pathological sex differences in 3xTg‐AD mice. One possibility is that, although female 3xTg‐AD mice exhibited higher intake and increased PEMT activity, circulating choline levels remained low because more choline was utilized by adipose tissue for cell membrane synthesis, consistent with their increased body weight. While future studies will investigate the mechanisms underlying low circulating choline in this AD model, these reductions likely contribute to the metabolic and neurological dysfunctions observed in 3xTg‐AD mice.

Various studies utilizing the 3xTg‐AD mouse model only include females because of their predictable pathological development (Dennison et al. 2021; Barber et al. 2024; Oddo et al. 2003; Velazquez et al. 2017; Judd et al. 2024; Winslow et al. 2021). While we observed variability in pathological development in the present study, we also observed that 3xTg‐AD males had a high mortality rate throughout the study, with only 23% of them surviving to the end of the study compared to 52% of 3xTg‐AD females and 80% of NonTg males. The survival curves demonstrate sex‐specific patterns in mortality in the 3xTg‐AD model. Whereas 3xTg‐AD males exhibited a relatively abrupt decline in survival later in life, 3xTg‐AD females displayed a more gradual but notable decrease beginning around 6 months and extending through approximately 14 months. This period corresponds to the emergence and progression of AD‐related neuropathology in female 3xTg‐AD mice, which has been shown to occur earlier and more robustly than in males. Additionally, female 3xTg‐AD mice exhibit metabolic alterations, including impairments in glucose homeostasis, which may have further contributed to both reduced survival and behavioral outcomes (Dave et al. 2023; Winslow et al. 2021). Studies that include both sexes of 3xTg‐AD mice frequently report that males have shorter lifespans and poor health, and higher frailty than females (Dennison et al. 2021; Barber et al. 2024). 3xTg‐AD males have lower body weight and fat, which can increase the likelihood of disease development at later ages (Robison et al. 2023, 2020). They also show inflammation in their hypothalamus, impacting energy and glucose homeostasis (Robison et al. 2023, 2020). 3xTg‐AD males have more severe peripheral pathology than females, including progressive hepatic inflammation, splenomegaly, and elevated circulating inflammatory proteins (Barber et al. 2024; Robison et al. 2020, 2023). 3xTg‐AD males also exhibit high chronic immune system activation throughout their lifespan (Barber et al. 2024; Dennison et al. 2021). Although this upregulation in immune system function is thought to protect 3xTg‐AD males against the development of neuropathologies, chronic immune system activation results in long‐term systemic inflammation and is believed to contribute to their shorter life and poorer health spans (Barber et al. 2024; Dennison et al. 2021; Kapadia et al. 2018). We previously showed that lower circulating choline levels are correlated with higher inflammation (Judd et al. 2023). Further, liver dysfunction is a well‐established consequence of inadequate choline supply (Dave et al. 2023; Institute of Medicine 1998), suggesting that lower circulating choline levels in 3xTg‐AD may contribute to this peripheral dysfunction. Importantly, the PSEN1 M146V knock‐in mutation in this model is expressed ubiquitously, not just in the central nervous system (Velazquez et al. 2017; Oddo et al. 2003). PSEN1 mutations have been associated with peripheral dysfunctions, including alterations in metabolites (Natarajan et al. 2021) and impaired glucose tolerance (Vandal et al. 2015). PSEN1 expression is modulated by age and sex hormones; testosterone reduces PSEN1 expression in young, but not old male mice, while estrogen reduces PSEN1 in females regardless of age (Ghosh and Thakur 2008). Further, males and females are differently impacted by dietary variations; when 3xTg‐AD mice are placed on a high fat diet, females gain more weight, adiposity, and glucose intolerance, while males show increased diabetes markers and systemic inflammation (Robison et al. 2023, 2020). Together, this study and others illustrate that while 3xTg‐AD males are relatively spared from the neuropathologies that are characterized in females, aging exacerbates the peripheral effects of the mutations, which may ultimately contribute to their reduced lifespan.

In the IntelliCage, we observed genotype and sex differences across several phases of testing. Throughout the sessions, males engaged in more water‐seeking behavior, as measured through total visits and total licks. During the RT tasks with a variable pre‐cue and a 7 or a 1‐s cue, males made a higher proportion of correct responses than females. However, during PP, reversal and all the other RT tasks, males and females had a similar proportion of correct responses. Further, males showed impairment during Avoidance; they made more visits to the corner with an air puff than females. This highlights that males were more motivated by water‐seeking behavior than females and is consistent with motivational sex differences we had previously observed in the APP/PS1 mouse model of amyloidosis (Mifflin et al. 2021). These sex differences replicate findings from other behavioral paradigms that require similar cognitive domains; males often perform better in spatial learning and memory tasks (Zorzo et al. 2024; Sutcliffe et al. 2007; Mifflin et al. 2021), while females do better on avoidance tasks (Dalla and Shors 2009). Additionally, during RT tasks, 3xTg‐AD mice were more likely to respond prematurely and less likely to respond correctly than NonTg mice. This aligns with our recent report (Judd et al. 2024) which showed that 3xTg‐AD mice display higher impulsivity during RT tasks in the IntelliCage than NonTg mice. Interestingly, NonTg males showed a notable decline in body weight after IntelliCage testing. Given their greater initial weight, it is likely the experience of the IntelliCage contributed to their weight loss. While 3xTg‐AD males and their NonTg counterparts can be housed together in the IntelliCage without substantial fighting, they may have experienced the expanded group housing over 46 days as a chronic stress situation, which is known to result in weight loss in males (Judd et al. 2025). Further, there was widespread variation in the endpoint weight of the NonTg males, suggesting that they may have been differentially stressed by the environment, perhaps based on the dominance hierarchy (Tamashiro et al. 2007). Notably, a large part of the premature deaths in the 3xTg‐AD occurred during this duration, suggesting that the stress of the expanded group housing may have exacerbated their peripheral pathologies, resulting in mortality. This possibility should be considered in future studies using the IntelliCage with male mice and monitoring for dominance hierarchies may reveal differences in behavioral and health outcomes. Together, this data characterizes sex differences in the 3xTg‐AD mouse in the IntelliCage and shows that the IntelliCage is a useful tool to interrogate sex differences in 3xTg‐AD mice on spatial learning and memory, attentional reaction time, and avoidance tasks.

Prior studies have shown that female 3xTg‐AD mice exhibit lower circulating plasma choline compared to NonTg mice, and that this deficit is further exacerbated by dietary choline deprivation, accelerating Aβ pathology and tau hyperphosphorylation (Judd et al. 2023). In humans, reduced circulating choline levels have similarly been associated with pathological progression in AD (Judd et al. 2023), and accumulating evidence indicates that inadequate choline intake increases the risk of developing the disease (Yuan et al. 2022). Importantly, low circulating choline may drive AD pathogenesis by enhancing both systemic and central inflammatory responses, thereby compromising neuronal integrity and promoting immune‐mediated mechanisms that contribute to disease progression. To that end, lower choline intake is associated with elevated levels of pro‐inflammatory cytokines, such as IFN‐γ (Baker et al. 2023; Winslow et al. 2025), IL‐1B (Huang et al. 2025; Baker et al. 2023; Winslow et al. 2025), IL‐2 (Winslow et al. 2025), IL‐6 (Huang et al. 2025), IL‐12 p70 (Winslow et al. 2025), and TNF‐α (Huang et al. 2025; Baker et al. 2023; Winslow et al. 2025). Lower choline levels are also associated with higher levels of anti‐inflammatory cytokines IL‐5 and IL‐13 (Winslow et al. 2025) and dysregulation of inflammatory response mechanisms (Dave et al. 2023). 3xTg‐AD mice have elevated inflammation compared to NonTg and, while accelerated inflammation in the males contributes to their peripheral pathology, evidence suggests chronic low‐grade inflammation also contributes to AD pathological burden (Winslow et al. 2025; Dave et al. 2023; Dennison et al. 2021; Barber et al. 2024; Giunta et al. 2008). Higher levels of IL‐1β, IL‐6, and TNF‐α are linked with worse pathological burden (Judd et al. 2023; Chen et al. 2024; Rani et al. 2022). We have also shown that higher circulating choline levels correspond with lower levels of the pro‐inflammatory cytokine TNF‐α (Judd et al. 2023). Additionally, we and others have shown that choline supplementation reduces disease‐associated microglial activation, lowers Aβ burden, and rescues cognitive deficits in APP/PS1 mice (Velazquez et al. 2019; Huang et al. 2025). Taken together, this suggests that lower circulating choline in the 3xTg‐AD mouse may promote inflammation and thereby facilitate pathological development, and also highlights the potential beneficial effects of additional choline in diet.

This study highlights the neuropathological sexual dimorphism observed in the 3xTg‐AD mouse model. 3xTg‐AD mice had lower circulating choline across the lifespan, including prior to the development of extensive pathology. Lower circulating choline levels may result in sex‐specific dysfunctions, contributing to peripheral disease in males and neuropathology in females. Given that circulating choline levels did not parallel consumption differences or PEMT protein levels, these data suggest that dietary intake and endogenous production by PEMT do not fully explain blood choline level differences. Rather, consuming the recommended daily intake amount of choline may not fully compensate for individual genetic differences in choline demand in 3xTg‐AD mice. Together, this work reveals a novel, lifelong reduction in circulating choline levels despite adequate intake in 3xTg‐AD mice, which may differentially contribute to AD pathology in females and frailty in males, underscoring the potential need for individualized choline recommendations to improve health outcomes.

## Author Contributions

J.M.J. and F.M.: Equal contribution to writing, data analysis, and animal experiments. WW: ELISA, statistical analysis, and helped write the manuscript. S.T.: Animal experiments, data analysis, and helped write the manuscript. J.T.: Animal experiments, data analysis, and helped write the manuscript. R.V.: Experimental design, animal studies, and helped write and edited the manuscript. All authors read and approved the final manuscript.

## Funding

This work was supported by the National Institutes of Health (R01AG062500) and Arizona State University, Edson Seed Grant.

## Conflicts of Interest

Ramon Velazquez is a research advisor for Performance Lab LLC. No funds from Performance lab were used for the study.

## Supporting information


**Figure S1:** During the IntelliCage adaptation phases, all mice acquired the rules of the task, and males were more water‐motivated than females. (A) Illustration of the IntelliCage system (B) and operant corners of the IntelliCage. (C) Timeline of behavior test tasks in the IntelliCage. During free adaptation, (D) total visits decreased, and (E) total licks increased across days. During door adaptation, (F) total visits decreased, and (G) total licks increased across days. During nose poke adaptation, (H) total visits decreased across days, and males made more total visits. (I) Total licks during nose poke adaptation (J) increased from Day 1 to Day 2 in females only. (K) Males made more visits with ≥ 1 lick than females. During water restriction adaptation, males made more (L) total visits, (M) total licks, (N) visits with ≥ 1 lick, and (O) visits during water access than females. Data are reported as means ± SEM *p < 0.05, **p < 0.01, ***p < 0.001, ****p < 0.0001.
**Figure S2**: Full uncropped Western blots of the PEMT protein and loading control GAPDH. A total of two Western blots were run, each including a shared between blot control (bbc) sample to normalize and account for variability between blots. NonTg male = 8, NonTg female = 13, 3xTg‐AD male = 6, and 3xTg‐AD female = 12.

## Data Availability

The data that support the findings of this study are available on request from the corresponding author. The data are not publicly available due to privacy or ethical restrictions.
