# Supplementary material for: The 3xTg‐AD Mouse Model of Alzheimer's Disease Exhibits Lifelong Reductions in Circulating Choline Despite Adequate Dietary Intake, With Sex‐Specific Neuropathological and Behavioral Phenotypes
Source: Aging Cell. 2025 Dec 29;25(1):e70330. doi: 10.1111/acel.70330 (PMC12745837; doi:10.1111/acel.70330)

**Supplemental Figure 1: During the IntelliCage adaptation phases, all mice acquired the rules of the task, and males were more water-motivated than females.** **(A.)** Illustration of the IntelliCage system **(B.)** and operant corners of the IntelliCage. **(C.)** Timeline of behavior test tasks in the IntelliCage. During free adaptation, **(D.)** total visits decreased, and **(E.)** total licks increased across days. During door adaptation, **(F.)** total visits decreased, and **(G.)** total licks increased across days. During nose poke adaptation, **(H.)** total visits decreased across days, and males made more total visits. **(I.)** Total licks during nose poke adaptation **(J.)** increased from day 1 to day 2 in females only. **(K.)** Males made more visits with ≥1 lick than females. During water restriction adaptation, males made more **(L.)** total visits, **(M.)** total licks, **(N.)** visits with ≥1 lick, and **(O.)** visits during water access than females. Data are reported as means ± SEM**p* < 0.05, ***p* < 0.01, ****p* < 0.001, *****p* < 0.0001.


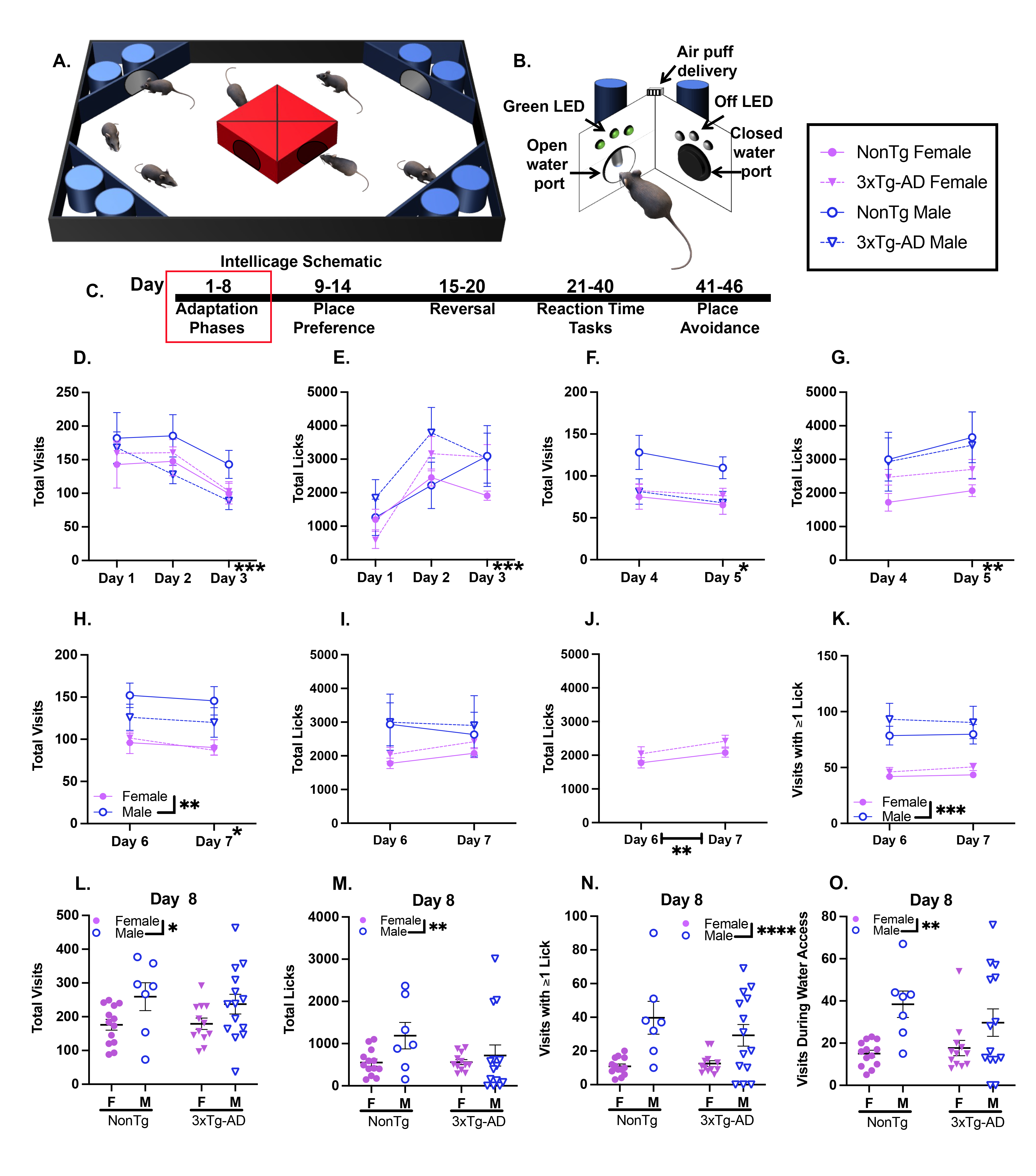


**Supplemental Figure 2:** **Full uncropped western blots of the PEMT protein and loading control GAPDH.** A total of two Western blots were run, each including a shared between blot control (bbc) sample to normalize and account for variability between blots. NonTg male = 8, NonTg female = 13, 3xTg-AD male = 6, 3xTg-AD female = 12.


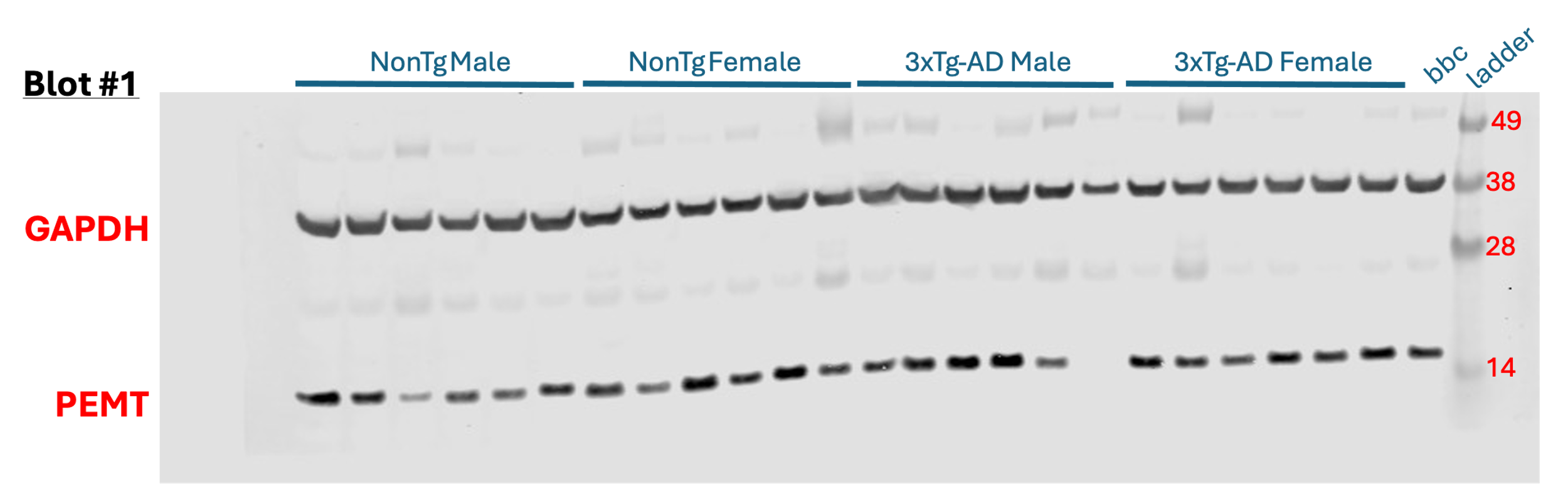


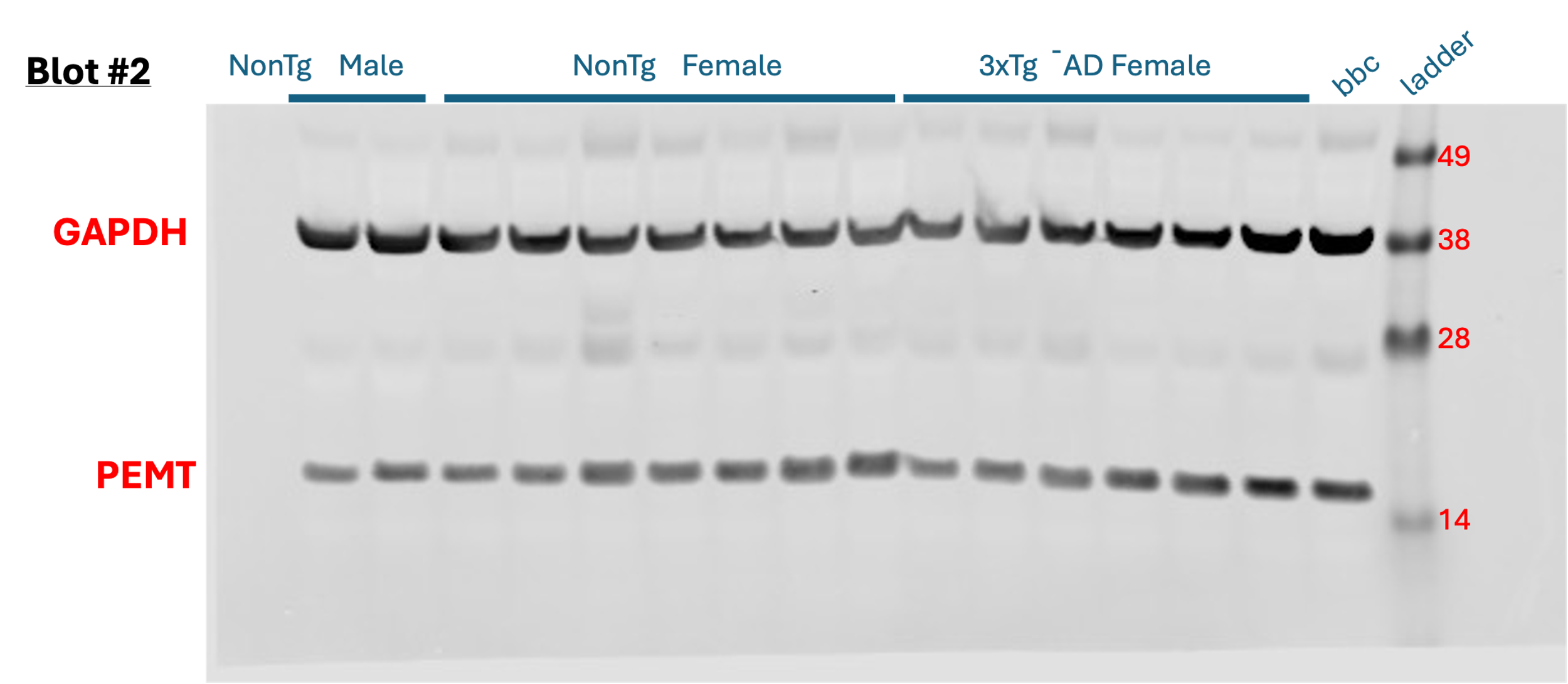

Supplement: Supplementary file 1 — Figure S1: During the IntelliCage adaptation phases, all mice acquired the rules of the task, and males were more water‐motivated than females. (A) Illustration of the IntelliCage system (B) and operant corners of the IntelliCage. (C) Timeline of behavior test tasks in the IntelliCage. During free adaptation, (D) total visits decreased, and (E) total licks increased across days. During door adaptation, (F) total visits decreased, and (G) total licks increased across days. During nose poke adaptation, (H) total visits decreased across days, and males made more total visits. (I) Total licks during nose poke adaptation (J) increased from Day 1 to Day 2 in females only. (K) Males made more visits with ≥ 1 lick than females. During water restriction adaptation, males made more (L) total visits, (M) total licks, (N) visits with ≥ 1 lick, and (O) visits during water access than females. Data are reported as means ± SEM *p < 0.05, **p < 0.01, ***p < 0.001, ****p < 0.0001. Figure S2: Full uncropped Western blots of the PEMT protein and loading control GAPDH. A total of two Western blots were run, each including a shared between blot control (bbc) sample to normalize and account for variability between blots. NonTg male = 8, NonTg female = 13, 3xTg‐AD male = 6, and 3xTg‐AD female = 12. [file ACEL-25-e70330-s001.docx]
